# Total scattering reveals the hidden stacking disorder in a 2D covalent organic framework†

**DOI:** 10.1039/d0sc03048a

**Published:** 2020-07-08

**Authors:** Alexander M. Pütz, Maxwell W. Terban, Sebastian Bette, Frederik Haase, Robert E. Dinnebier, Bettina V. Lotsch

**Affiliations:** Max Planck Institute for Solid State Research Heisenbergstrasse 1 70569 Stuttgart Germany b.lotsch@fkf.mpg.de m.terban@fkf.mpg.de; Department of Chemistry, University of Munich (LMU) Butenandtstrasse 5-13 81377 Munich Germany; Exzellenzcluster E-conversion Lichtenbergstrasse 4a 85748 Garching Germany

## Abstract

Interactions between extended π-systems are often invoked as the main driving force for stacking and crystallization of 2D organic polymers. In covalent organic frameworks (COFs), the stacking strongly influences properties such as the accessibility of functional sites, pore geometry, and surface states, but the exact nature of the interlayer interactions is mostly elusive. The stacking mode is often identified as eclipsed based on observed high symmetry diffraction patterns. However, as pointed out by various studies, the energetics of eclipsed stacking are not favorable and offset stacking is preferred. This work presents lower and higher apparent symmetry modifications of the imine-linked TTI-COF prepared through high- and low-temperature reactions. Through local structure investigation by pair distribution function analysis and simulations of stacking disorder, we observe random local layer offsets in the low temperature modification. We show that while stacking disorder can be easily overlooked due to the apparent crystallographic symmetry of these materials, total scattering methods can help clarify this information and suggest that defective local structures could be much more prevalent in COFs than previously thought. A detailed analysis of the local structure helps to improve the search for and design of highly porous tailor-made materials.

## Abstract 2

With total scattering methods and stacking fault simulations, we observe previously predicted random local layer offsets in a COF, which are typically disguised by the apparent crystallographic symmetry but strongly influence properties.

## Graphical abstract

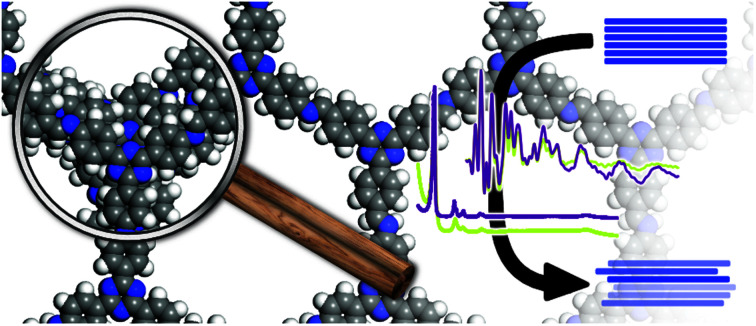

## Introduction

Covalent organic frameworks (COFs) are crystalline, porous polymers assembled from building blocks in a reticulating reaction. Depending on the geometry of the linkers, they form either 2D sheets, where layers stack *via* dispersive forces, or 3D covalently connected frameworks. COFs possess well-defined micro- and mesoporous structures, where pore size, shape, topology, and the distribution of readily accessible active functional sites are defined with molecular precision.^1^ Applications such as small molecule separation, capture and storage, (opto-)electronics, and catalysis are particularly promising due to the tunability of these properties.^2,3^

**Fig. 1:**
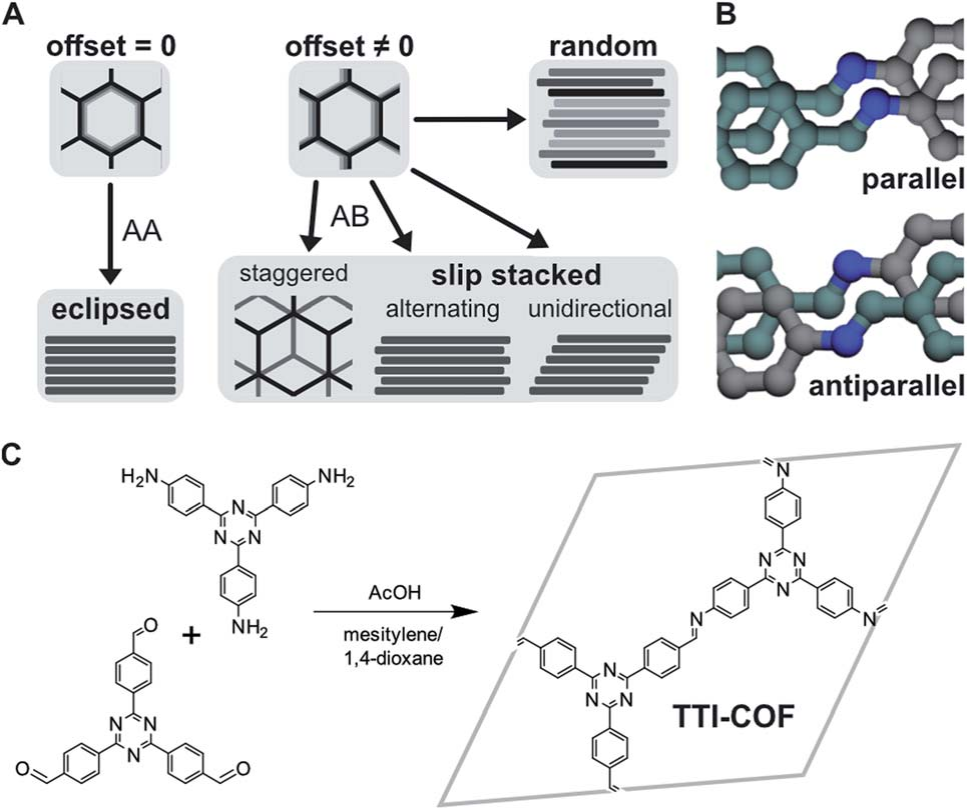
(A) Different stacking modes, depending on magnitude and direction of offset between neighboring layers. Random stacking can occur along multiple directions. (B) Parallel and antiparallel orientation of imine bonds (grey: amine carbon, cyan: aldehyde carbon, blue: nitrogen). (C) Formation of TTI-COF by condensation of aldehyde and amine.

The sheets that comprise 2D COFs can stack in various ways, as shown in Fig. 1A. The offset between neighboring layers in the *a*–*b* plane can be formally equal or unequal to zero, resulting in eclipsed stacking or slipped stacking.^4,5^ In the latter case, alternating and unidirectional slip stacking are differentiated, where the offset occurs in the same or alternating directions. Staggered stacking represents a special case of AB-type slip stacking, where the offset is such that the vertex of one layer is above the pore of another, similar to graphite.^6^ The symmetry of these stacking motifs decreases in order from eclipsed, staggered, alternating, to unidirectional. Other scenarios could involve different combinations of these motifs or fully random stacking, which is more difficult to characterize due to the lack of translational order.

The geometry of specific linker molecules can generate ordered layer stacking by offering a templating effect during the growth of new layers,^7–11^ as thermodynamics generally govern the arrangement of linker and small oligomer molecules. On the other hand, because the stacking energy is too high to be overcome at typical reaction temperatures,^4,12–14^ layer aggregation, as opposed to linker and oligomer adsorption, is effectively irreversible, which results in stacking disorder in most COFs. The in-plane disorder can also be caused by flexible linkers and influences stacking interactions, leading to further out-of-plane disorder.^15^ It therefore follows that understanding the local structure of a given COF is vital, because properties such as pore geometry, accessibility of functional sites, interaction with guest molecules, (opto-)electronic properties, and surface states in the pore significantly depend on the layer stacking.^4,7,16,17^ The prevalent notion is that most COFs must still have a local structure dominated by a layer offset, despite apparent high symmetry and eclipsed stacking.^5,8,18–24^ Techniques that offer insight into the local order and stacking of COFs are thus instrumental in understanding and developing novel materials for particular applications in a directed manner.

Here, we directly investigate the local symmetry of two related imine COFs by a combination of X-ray diffraction, stacking fault simulations, spectroscopy, electron microscopy, and physisorption analysis, and show how ordered and disordered slip stacking manifests. We evaluate short- and long-range order in terms of defect abundance, stacking, and morphology and show that in fact, random slip stacking is easily misinterpreted as apparent eclipsed stacking.

## Results and discussion

TTI-COF was synthesized by condensation of the corresponding tritopic aldehyde and amine under solvothermal conditions in a mesitylene/1,4-dioxane 1 : 1 mixture, catalyzed by aqueous 6 M acetic acid. We prepared two differently stacked forms: **HT** at high temperature, *i.e.*, 120 °C,^25^ and **LT** at room temperature. Fig. 1C shows the linkers and simplified reaction scheme, where the imine-linked layer is represented by the hexagonal unit cell.

### Spectroscopy

**Fig. 2:**
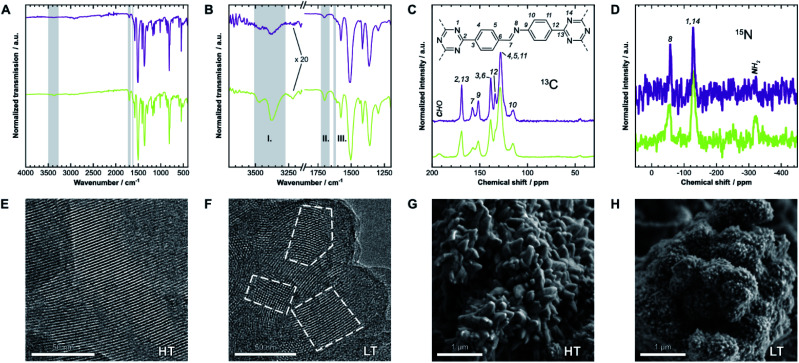
(A) Fourier-transform infrared (FT-IR) spectra of **HT** (purple) and **LT** (green). (B) Detail view of two characteristic regions of the spectrum. Gray regions highlight bands generated by amine (I), aldehyde (II), and imine groups (III). (C) ^13^C and (D) ^15^N solid-state nuclear magnetic resonance (ssNMR) spectra with assignments.^21^ Gray regions highlight signals from residual aldehyde and amine groups, respectively. (E) Transmission electron micrographs of **HT** and (F) of **LT**. Some crystallites are highlighted to demonstrate the size difference. (G) Scanning electron micrographs of **HT** and (H) of **LT**, which also show significantly different morphologies.

We first have to confirm the chemical identity and formation of the COF. Fourier-transform infrared (FT-IR) spectra presented in Fig. 2A and B show a reduction of the characteristic amine (I) bands at 3473 cm^−1^ and 3379 cm^−1^, and aldehyde (II) bands at 1700 cm^−1^, when compared to the starting materials (Fig. SI-1C and D†). Instead, a new band at 1624 cm^−1^ (III), which is weak in this COF,^25^ indicates the formation of imine linkages. Weak residual bands corresponding to amine and aldehyde groups, which are significantly stronger for **LT**, are explained by terminal functional groups and trapped linker molecules. Lastly, the bands at 1509 cm^−1^ and 1365 cm^−1^ in **HT** are characteristic for triazines,^26^ but notably shifted by 6 cm^−1^ to lower frequencies in **LT**, which may suggest different interlayer interactions in the two samples.

The local structure of the COFs was also investigated by ^13^C and ^15^N solid-state nuclear magnetic resonance spectroscopy (ssNMR), shown in Fig. 2C and D. Signals of the corresponding carbon at 158 ppm (^13^C) and nitrogen at −58 ppm (^15^N) indicate the formation of the imine bond. On comparing the spectra of both samples, two main differences become apparent: similar to FT-IR spectroscopy, signals from residual amine and aldehyde groups are only present in **LT** at −322 ppm (^15^N) and 192 ppm (^13^C), respectively. These signals suggest more residual surface groups. All NMR signals are also significantly broader for **LT**, which indicates a wider distribution of local chemical environments compared to **HT**.

### Electron microscopy

The hexagonal pore structure and one-dimensional pore channels of the COF are observed in transmission electron microscopy (TEM), as shown in Fig. 2E and F. Using fast Fourier transforms and intensity profiles of these micrographs, we determined the periodicity of these features (see Fig. SI-2†). The measured values correspond to the *d*-spacing of the 100 and 110 reflections, 22 Å and 13 Å, respectively, which also match results obtained from X-ray diffraction (see below). The micrographs also show that the average crystallite size in **HT** is significantly larger than in **LT**. Crystallites of over 100 nm can be observed in **HT**, while many crystallites with sizes of under 50 nm are prevalent in **LT** (see Fig. SI-4† for additional representative micrographs). Smaller domain sizes also account for the increased occurrence of free aldehydes and amines in IR and ssNMR spectroscopy for **LT** over **HT** owing to the increased surface-area-to-volume ratio. Similar results can be inferred from scanning electron microscopy (SEM), shown in Fig. 2G and H. Both samples present themselves with a dendritic cauliflower-like morphology but with much smaller aggregated particles in **LT**.

### Sorption analysis

**Fig. 3:**
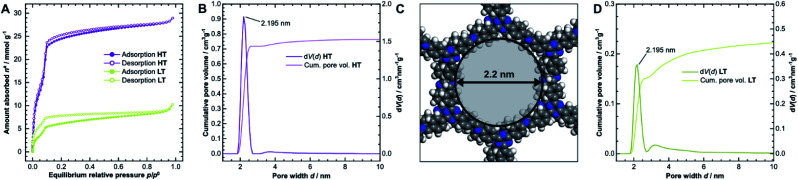
(A) Physisorption isotherms of **HT** (purple) and **LT** (green), collected using argon at 87 K. (B) Cumulative pore volume and pore size distribution (PSD) for **HT** as calculated by quenched solid density functional theory (QSDFT) using the adsorption branch kernel. (C) The calculated pore size matches the diameter determined from the optimized unidirectionally slip-stacked structure of TTI-COF (see Fig. SI-8F,† hydrogens added) by measuring the distance of opposing atoms. (D) Cumulative pore volume and PSD for **LT**.

The porosity of the COFs was analyzed *via* argon physisorption, and the resulting isotherms are presented in Fig. 3A. After an initial monolayer-multilayer adsorption step and pore condensation, a saturation plateau dominates the isotherms over *p*/*p*^0^ = 0.10.^27^ These features are characteristic for type IV(b) isotherms, which are common for mesoporous materials.^28^ The BET areas were determined to be 1308 m^2^ g^−1^ for **HT** and only 338 m^2^ g^−1^ for **LT**. This trend is also reflected by the total pore volume as determined from the maximum amount of gas absorbed at the saturation pressure *p*^0^. We measured total pore volumes of 0.825 cm^3^ g^−1^ for **HT** and 0.292 cm^3^ g^−1^ for **LT**. Such significant differences in BET areas and pore volumes indicate that most of the internal surface area in **LT** is not accessible to the adsorbate because of pore blockage.

We also observed that **HT** exhibits only a small amount of hysteresis, which is much more pronounced in **LT**. Since the hysteresis extends to very small relative pressures, physical effects, such as percolation effects, cavitation, or capillary condensation cannot be its sole cause.^28–31^ Instead, it is probably caused by severely limited diffusion of the adsorbate through the porous material. The stiff geometry of the linker molecules and strong interlayer interactions ideally lead to uniform pores in COFs. Stacking faults can, however, generate constrictions at the pore entrances or within the channel, which hinder diffusion pathways and trap linker or oligomer molecules. Due to the hysteresis, the pore size distribution (PSD) was determined from the adsorption branch.^29–32^ Quenched solid density functional theory (QSDFT) gives average pore widths of 2.2 nm for **HT** (Fig. 3B) and **LT** (Fig. 3D). This dimension matches the diameter obtained from the optimized structure of TTI-COF, illustrated in Fig. 3C. In **LT**, however, the PSD is wider, which indicates a more disordered pore structure.

### Diffraction

**Fig. 4:**
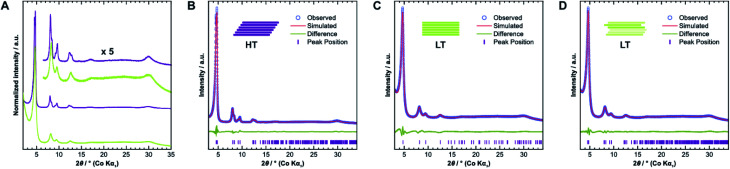
(A) Comparison of X-ray diffraction patterns of **HT** (purple) and **LT** (green) collected with Co Kα_1_ radiation. (B) Diffraction pattern of **HT** with best-obtained fit by Rietveld refinement assuming unidirectional slip stacking. (C) Diffraction pattern of **LT** with best-obtained fit by Rietveld refinement assuming eclipsed stacking and (D) random stacking.

We confirmed the crystallinity of both samples by X-ray powder diffraction (XRPD), see Fig. 4A. **HT** exhibits narrower Bragg peaks and additional peak splitting on the first four reflections. The peak broadening of **LT** is, however, particularly pronounced in the stacking reflections at 30° 2*θ*. Anisotropic crystallite size broadening and microstrain due to local disorder can both result in peak broadening, but contributions from these effects cannot be distinguished easily for this class of materials, because of the typically low quality of diffraction data. In earlier work, our group showed that the peak splitting results from symmetry reduction caused by a unidirectionally slip-stacked structure. Density functional theory calculations found an optimum stacking offset of around 1.6 Å and showed that an antiparallel linker orientation is preferred.^25^ When two tritopic linkers are used, they can either stack in a parallel or antiparallel fashion, as shown in Fig. 1B. These cases lead to imine linkages oriented in the same or opposite directions, respectively.^4^

We used the unidirectional slip-stacked, antiparallel structure model as a basis for the Rietveld refinement of **HT**.^25^ Rietveld refinements were performed using TOPAS-Academic v6, taking into account the instrumental profile and crystallite size and microstrain broadening.^33^ The resulting fit, shown in Fig. 4B, is of good quality and describes the experimentally observed pattern reasonably well. The unidirectional stacking of layers causes a reduction of the symmetry, which results in the observed peak splitting. In contrast, using an eclipsed structure model returns a poor Rietveld fit (see Fig. SI-6A†) because it cannot describe the additionally observed Bragg peaks. Consequently, Rietveld refinements showed that **LT** is best described by the eclipsed rather than slip-stacked structure, as shown in Fig. 4C, albeit with a much smaller crystallite size as observed by TEM. However, the refinement also indicates a severe amount of strain in **LT** compared to **HT**, which suggests that the local structure of this material is not well-described by the eclipsed stacking motif.

**Fig. 5:**
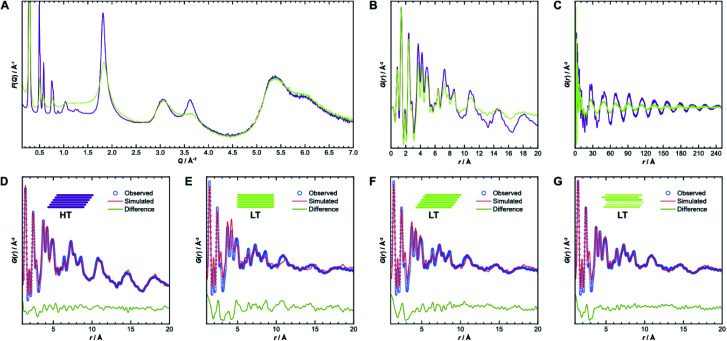
(A) Comparison of reduced total scattering structure functions *F*(*Q*) for **HT** (purple) and **LT** (green) collected using synchrotron radiation. (B) Pair distribution functions derived thereof up to 20 Å and (C) up to 250 Å. (D) Best obtained PDF fits over 1–20 Å from structure refinements of **HT**, assuming unidirectionally slipped stacking, and (E) of **LT**, assuming eclipsed, (F) unidirectionally slipped, or (G) randomly slipped stacking.

To gain further insight into the samples' atomic-scale details, we performed pair distribution function (PDF) analysis.^34–38^ We collected total scattering data using synchrotron radiation, which was first converted into the reduced total scattering structure function *F*(*Q*) (Fig. 5A, *cf.* ESI Methods section†), with the elastic scattering momentum transfer *Q* = 4π sin(*θ*)*λ*^−1^, using the PDFgetX3 algorithm within xPDFsuite.^36,39,40^ A considerable reduction in the intensity of the peaks located at 1.8 Å^−1^ and 3.6 Å^−1^ is observed in **LT** compared to **HT**, while the peak at 3.0 Å^−1^ is the same for both samples. The two peaks with reduced intensity contain strong contributions from the 002 and 004 reflections, respectively, and systematic broadening and intensity reduction here could be associated with both reduction in crystallite size along the stacking direction as well as stacking disorder. The patterns are, however, nearly identical above 4.0 Å^−1^. The high-*Q* scattering and the peak at 3.0 Å^−1^ result mostly from in-plane components, indicating that the individual layers remain conformationally consistent between both samples, which could also be confirmed by simulations performed using the software XISF (see Fig. SI-7†).^41^

The pair distribution function *G*(*r*) is obtained by Fourier transformation of *F*(*Q*). Here, *G*(*r*) can be roughly divided into three length scales: (I) very sharp peaks at short distances under 6.0 Å, which correspond to specific atom-pair distances within the layers, (II) intermediate frequency peaks, which are associated with the layer stacking (both Fig. 5B), and (III) broad, low-frequency peaks, which result from the COF pores (Fig. 5C). The frequencies of the latter two components match the 002 and 100 reflections, with *d*-spacings of 3.7 Å and 22 Å, respectively. The low-frequency component associated with the pore structure dominates both PDF signals over long distances above 200 Å (see Fig. SI-10A and B†). The intensity of these peaks is lower in **LT** than in **HT**, which suggests some combination of increased disorder in the layer offset, more distortions of the pore shape, trapped pore content, and decreased crystallite size. By truncating the reduced total structure function to *Q* values above 1.5 Å^−1^, we were also able to isolate the stacking component of the PDFs for **HT** and **LT** (see Fig. SI-10C and D†). The coherence lengths of these signals are roughly 70 Å and 50 Å for **HT** and **LT**, respectively, showing a relatively lower degree of order in the stacking direction.

Structure refinements to the PDF data using different models were performed in PDFgui, with experimental broadening and damping from finite *Q*_max_ and instrumental profile effects fixed.^37^ Structural and thermal effects were accounted for in the lattice parameters, atomic displacement parameters (ADPs), and low-*r* peak sharpening by correlated motion corrections (see ESI for more details†). The structure model with unidirectional slip stacking gave a good Rietveld fit for **HT** and likewise returned a good PDF fit over 1 Å to 20 Å, as shown in Fig. 5D. Sharp peaks corresponding to short interatomic distances within a single layer and broad peaks due to interlayer interactions can both be well described using ADPs with *U*_11_ = *U*_22_ within the layer and separately refined *U*_33_ for the out-of-plane distances.^42^ When the stacking orientations are not well described in the model, *U*_33_ tends toward higher values to broaden interlayer atom-pair correlations. We also compared models with eclipsed stacking and both antiparallel and parallel imine orientations (see Fig. SI-8 and SI-9†). In all cases, in-plane ADPs were low, indicating a good description of an ordered layer structure, but the stacking was not well described by the eclipsed models. An antiparallel, rather than parallel, imine orientation, showed better agreement with the experimental data, which corroborates the preference for antiparallel packing.^25^

While the lack of peak splitting suggests an eclipsed structure for **LT**, the high strain parameters derived from Rietveld refinements and the similarity of the PDF signals of **HT** and **LT** over short and intermediate-range distances (*cf.*Fig. 5B) point toward a more slipped local layer relationship instead. Indeed, Fig. 5E shows that while the intralayer contributions can still be described reasonably well by an eclipsed structure model, the peak positions corresponding to the layer stacking over short and intermediate distances do not match the experimental data. We also observe high ADPs in the stacking direction. We thus can assume that the layers in **LT** are slipped relative to each other, as would be thermodynamically more favorable and as attested to in **HT**.^20,43^ Indeed, using a unidirectionally slip-stacked structure to fit the local structure in the PDF improves the result, as seen in Fig. 5F. There is, however, still a mismatch between the observed and simulated peak positions above 10 Å, and this model conflicts with the high apparent symmetry of **LT** seen in XRD. To resolve these discrepancies and increase understanding of the overall stacking, we performed stacking fault simulations.

### Stacking fault simulations

The absence of Bragg peak splitting and apparent hexagonal symmetry in **LT** seem to suggest apparent zero offset between the layers. However, analysis of the local structure shows slipped stacking between neighboring layers, which is energetically favored.^4,43–47^ We also observe a generally high amount of disorder and strain in **LT** with complementary spectroscopy and diffraction methods. These seemingly conflicting findings can be resolved by random translational disorder from layer to layer, which would express itself in the same high-symmetry diffraction pattern as eclipsed stacking. The peak shapes observed in the diffraction pattern for **LT** further suggests interlayer disorder.^48^ Different stacking scenarios were investigated using *DIFFaX* to check consistency with the experimental data (see ESI† for more information and input file).^49^

**Fig. 6:**
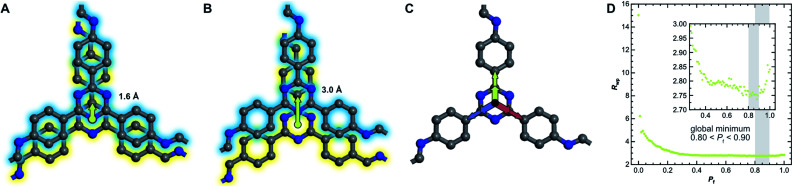
(A) Two neighboring layers of TTI-COF with antiparallel imine orientation (amine, blue, stacked on aldehyde, yellow) offset by 1.6 Å and (B) by 3.0 Å relative to each other with stacking vectors represented by green arrows. (C) Offset can occur in three directions, rotated by 120°, due to the trigonal symmetry of the linker molecules. (D) The quality of the Rietveld fits as described by the weighted profile *R*-factor (*R*_wp_) depending on the stacking fault probability *P*_f_. The global minimum between 0.80 and 0.90 is highlighted.

We then investigated this disorder in **LT** by Rietveld refinement, where we used a supercell approach,^33^ averaging the calculated diffraction patterns of 300 supercells containing 200 layers each. Starting from the optimized layer structure of **HT** with an antiparallel orientation of the imines, we defined two different layer offsets where neighboring layers are slipped along the direction of a pore wall. When the projected distance between two triazine ring centers is 1.6 Å (Fig. 6A), one triazine nitrogen atom is directly above the center of the previous ring. When the distance is increased to 3.0 Å (Fig. 6B), the nitrogen atom overlaps with the previous layer's triazine carbon. Due to the symmetry of the building blocks, both stacking vectors can be rotated by 120° and 240° along the layer plane to create a total of six different stacking transitions, as illustrated by Fig. 6C.

Instead of describing the disorder with microstrain parameters, we built a faulting scenario with these six vectors, where each transition probability relates to the stacking fault probability *P*_f_ (see Table SI-3†). A grid search optimization was performed by iterating the probability in small increments, resulting in Fig. 6D.^50,51^ Even with only little random stacking (*P*_f_ < 0.10), the quality of the Rietveld fits of **LT** increases vastly as compared to the unfaulted model. We found the best agreement to the experimental diffraction pattern in the region where 0.80 < *P*_f_ < 0.90, with a global minimum at *P*_f_ = 0.83, representing a complete loss of ordered stacking and almost equal probabilities for all slip-stacking transitions. Peak splitting is predicted based on the calculated peak positions. However, due to the random directionality of the slip stacking, only single broad peaks are observed for the *hk*0 reflections, which results in the observed apparent high symmetry.

We also refined the experimental PDF data of **LT** with structural models suited to simulate a randomly stacked material. We built hexagonal supercells from between two and six antiparallel layers that could translate freely in the *a* and *b* directions during PDF refinements. With an increasing number of layers, the quality of the fits improved significantly (see Fig. SI-18†), which was mainly reflected by the lower out-of-plane ADP. The result of the refinement with six layers is presented in Fig. 5G and shows how well random stacking can describe the stacking component for *r* > 10 Å. We estimated the average stacking offset by refining the PDF in the range of neighboring layers, *i.e.*, *r* < 6 Å. The resulting value of 1.63 Å fits very well with the energetically preferred lateral offset for COFs, which has been calculated as 1.7 Å.^4,25,43–47^

This slip-stacking motif is not exclusive to 2D polymers, but can also be found in aromatic molecular systems, both experimentally and theoretically.^52–56^ The attractive interactions between stacked aromatic rings are commonly attributed to interactions between π electrons. Instead, electrostatic attraction between the edge and face of aromatic quadrupoles accounts for the offset stacking that is predominant in single-crystal structures of aromatic molecules.^57–59^ It can be assumed that the high stacking energy in COFs results from similar interlayer interactions. These results indicate that offset stacking might be ubiquitous in COFs even when eclipsed stacking is assumed.

## Conclusions

We have confirmed and modelled layer stacking disorder in a low- and high-temperature variant of an imine-based COF by Rietveld refinement and PDF analysis combined with stacking fault simulations. A high amount of terminal groups and disorder as suggested by physisorption and electron microscopy point to insufficient error correction, which prohibits the growth of large crystallites or an ordered layer structure. On the other hand, the reduced synthesis temperature allowed access to a different, kinetically trapped stacking motif in TTI-COF with higher apparent symmetry due to random average layer offsets of 1.6 Å. We therefore suggest that the synthesis temperature—and with it, crystallite size, amount of terminal groups, and layer connectivity—should be considered as a variable with which the stacking motif may be adjusted in 2D polymers.

Thus, we showed that the assignment of an eclipsed structure can be an oversimplification of the true local environment, as is indicated by the unfavorable energetics associated with these arrangements. We propose that many COFs reported as eclipsed structures very likely also feature random offset stacking motifs. X-ray diffraction data obtained from COFs is typically of lower quality than that of related materials, such as molecular organic crystals or metal organic frameworks, with much broader and also fewer Bragg peaks. The structure model obtained from such low-quality data is consequently less reliable, especially concerning the local order, which is instead often inferred from the linker geometry and structure modelling based on molecular mechanics or density functional theory calculations.^5^ We suggest then that structures inferred solely from pattern indexing or Rietveld fitting to low-quality data should be strictly interpreted in the crystallographic sense as *average* structures. In the absence of detailed structural insights into the stacking geometry, utmost care should be exercised when deriving structure-property relationships. Instead, by using the techniques mentioned above, additional information about the local structure can be extracted and help determine a more detailed picture of the atomic-level structure and stacking motifs present in a given COF.

To conclude, structural interpretations and properties calculated based on a purely crystallographic, *i.e.*, average, view of these structures can be unreliable, which can result in the misinterpretation of the inherent properties of COFs. This has been demonstrated for a wide range of materials such as perovskite photovoltaics,^60–62^ catalytic nanoparticles,^63,64^ exotic electronic materials,^65–67^ and more recently 2D polymer materials.^68,69^ Other complementary methods for tackling this problem are under active development.^70–74^ Thus, structural probes such as total scattering and PDF, as used here, could be valuable in obtaining a more distinct understanding of structuring pathways in 2D COFs and help to contextualize and optimize their functional behavior.

## Conflicts of interest

There are no conflicts to declare.

## Supplementary Material

SC-011-D0SC03048A-s001

SC-011-D0SC03048A-s002
