# Supplementary material for: Total scattering reveals the hidden stacking disorder in a 2D covalent organic framework
Source: Chem Sci. 2020 Jul 8;11(47):12647–54. doi: 10.1039/d0sc03048a (PMC8163241; doi:10.1039/d0sc03048a)
Supplement: SC-011-D0SC03048A-s001 [file SC-011-D0SC03048A-s001.pdf]

Electronic Supplementary Material (ESI) for Chemical Science.
This journal is © The Royal Society of Chemistry 2020

Electronic Supplementary Information (ESI) for

Total scattering reveals the hidden stacking disorder in a 2D covalent organic framework

Alexander M. Pütz, Maxwell W. Terban,* Sebastian Bette, Frederik Haase, Robert E. Dinnebier, and Bettina V. Lotsch*

This version of the ESI published 13/05/2021 replaces the previous version published 08/07/2020. The goodness-of-fit values have been added to Table SI-7, as well as to the corresponding fits in Figures SI-6, SI-8, SI-9, and SI-18.

Table of Contents

Methods SI-2
NMR and IR data SI-5
Electron microscopy SI-6
Physisorption analysis SI-8
Rietveld refinements SI-9
Reduced total scattering structure functions SI-9
Pair distribution function analysis SI-10
Rietveld and PDF stacking fault simulations SI-12
Additional data from Diamond Light Source SI-24
Summary of lattice parameters SI-25
References SI-26

Methods

Chemicals

2,4,6-Tris(4-aminophenyl)-1,3,5-triazine: To trifluoromethanesulfonic acid (3.51 mL, 39.2 mmol) and chloroform (10 mL) under argon was slowly added 4-aminobenzonitrile (1.18 g, 10.0 mmol) dissolved in chloroform (15 mL) at 0°C. After stirring for 16 h, the mixture was diluted with water (10 mL) and 2 M aqueous sodium hydroxide (20 mL) was added slowly to neutralize. The solids were filtered off and washed with water and diethyl ether. Drying in vacuo yielded the product as a yellow solid (265 mg, 0.750 mmol, 22.5 %). ^{1}H NMR (DMSO-d6, 400 MHz): δ_H/ppm 8.35 (d, 6H, ^3J = 8.8 Hz, arom.), 6.68 (d, 6H, ^3J = 8.8 Hz, arom.), 5.90 (s, 6H, NH_2). ^{13}C NMR (DMSO-d6, 101 MHz): δ_C/ppm 169.6, 153.0, 130.1, 122.9, 113.1.

2,4,6-Tris(4-bromophenyl)-1,3,5-triazine: Dried glassware was charged with 4-bromobenzonitrile (5.00 g, 27.5 mmol) under argon. Triflic acid (9.76 mL, 109 mmol) was added dropwise and the solution was stirred for 16 h. The reaction mixture was poured onto ice water (50 mL) and carefully neutralized with 2 M aqueous sodium hydroxide (55 mL). The precipitate was filtered off, washed with water and chloroform, and dried *in vacuo* at 60 °C to yield the crude product as a white solid (4.51 g, 8.44 mmol, 92.1 %). ^{1}H NMR ($CDCl_3$, 400 MHz): δ_H/ppm 8.60 (d, 6H, ^3J = 8.8 Hz, arom.), 7.71 (d, 6H, ^3J = 8.6 Hz, arom.) ^{13}C NMR ($CDCl_3$, 101 MHz): δ_C/ppm 135.0, 132.2, 130.7, 128.0.

4,4',4''-(1,3,5-Triazine-2,4,6-triyl)tribenzaldehyde: In dried glassware, 2,4,6-tris(4-bromophenyl)-1,3,5-triazine (1.00 g, 1.83 mmol) was dissolved in freshly distilled THF (95 mL). After cooling to -78 °C, *n*-butyllithium (2.5 M in hexanes, 2.42 mL, 6.04 mmol) was added dropwise. The mixture was stirred for 90 min, then 1-formylpiperidine (670 µL, 6.04 mmol) was added slowly. After warming to room temperature over 30 min, concentrated aqueous ammonium chloride (2.0 mL) was added and the mixture stirred for 10 min, then concentrated. The solids were dispersed in water/ethanol 1:1, filtered off and washed with water and ethanol. Drying *in vacuo* yielded the crude product as a white solid (0.414 g, 1.06 mmol, 57.9 %). ^{1}H NMR (DMSO-d6, 400 MHz): δ_H/ppm 10.20 (s, 3H, CHO), 8.98 (d, 6H, ^3J = 7.7 Hz, arom.), 8.20 (d, 6H, ^3J = 7.8 Hz, arom.). ^{13}C NMR (DMSO-d6, 101 MHz): δ_C/ppm 193.2, 170.7, 140.1, 139.3, 130.0, 129.6.

All other chemicals were obtained from commercial sources and used without further purification.

Synthesis

*High-temperature TTI-COF (**HT**):* 2,4,6-tris(4-aminophenyl)-1,3,5-triazine (86.5 mg, 0.244 mmol), 4,4',4''-(1,3,5-triazine-2,4,6-triyl)tribenzaldehyde (96.0 mg, 0.244 mmol), and a mesitylene/1,4-dioxane mixture (1:1 v/v, 19.2 mL) were added to a 20-mL Biotage microwave vial. The mixture was sonicated until finely dispersed and 6 M aqueous acetic acid (500 µL) was added. The vial was sealed and heated in an oil bath to 120 °C for 72 h. After cooling to room temperature, the solids were filtered off and washed thoroughly with ethanol, tetrahydrofuran, chloroform and dichloromethane. The COF was dried *in vacuo* to yield a yellow powder (103 mg, 60.8 %).

*Low-temperature TTI-COF (**LT**):* 2,4,6-tris(4-aminophenyl)-1,3,5-triazine (86.5 mg, 0.244 mmol), 4,4',4''-(1,3,5-triazine-2,4,6-triyl)tribenzaldehyde (96.0 mg, 0.244 mmol), and a mesitylene/1,4-dioxane mixture (1:1 v/v, 19.2 mL) were added to a 20-mL Biotage microwave vial. The mixture was sonicated until finely dispersed and 6 M aqueous acetic acid (500 µL) was added. The vial was sealed and heated in an oil bath to 120 °C for 72 h. After cooling to room temperature, the solids were removed and the COF was allowed to precipitate from the solution over 7 days. The COF was filtered off and washed thoroughly with ethanol, tetrahydrofuran, chloroform and dichloromethane. Drying *in vacuo* yielded a yellow powder (60.8 mg, 35.9 %).

Infrared spectroscopy

Infrared spectra were recorded in attenuated total reflection (ATR) geometry on a PerkinElmer Spectrum Two equipped with a diamond crystal. The background spectrum was measured separately and subtracted.

Nuclear magnetic resonance spectroscopy

Solution-state ^{1}H and ^{13}C nuclear magnetic resonance spectra were collected on a JEOL Resonance ECZ 400S spectrometer (B_0 = 9.40 T) at resonance frequencies of 400 MHz and 100.61 MHz, respectively. Chemical shifts for ^{1}H and ^{13}C were referenced against solvent residual signals. Solid-state ^{13}C and ^{15}N nuclear magnetic resonance experiments were performed on a Bruker Advance-III 400 MHz instrument (B_0 = 9.40 T) at a frequency of 100.61 MHz and 40.56 MHz, respectively. Chemical shifts for ^{13}C and ^{15}N were referenced to glycine

(δ_C = 176.46 ppm, 43.67 ppm; δ_N = -347.58 ppm).[1,2] Magic angle spinning with spinning rates ranging between 8 kHz and 12.5 kHz and cross-polarization were used in all experiments.

Transmission electron microscopy

Transmission electron microscopy was performed with a Philips CM30 ST (300 kV, LaB_6 cathode). The samples were suspended in isopropanol and drop-cast onto a lacey carbon film (Plano). The images were recorded with a CMOS camera F216 (TVIPS).

Scanning electron microscopy

Scanning electron microscopy secondary electron (SE) detector images were obtained on a Zeiss Merlin.

Physisorption analysis

Sorption measurements were performed on a Quantachrome Instruments Autosorb iQ with argon at 87K after activation at 120 °C for 12h. Analysis of the data was performed in Quantachrome® ASiQwin™. The pore size distribution was determined from argon isotherms using the quenched solid density functional theory (QSDFT) to account for surface heterogeneity.[3] The appropriate argon-carbon equilibrium transition (based on desorption branch) or adsorption branch kernels at 87 K were used based on a cylindrical pore model.[4] Due to hysteresis, accurate values can only be obtained from the adsorption branch of the isotherm.

Construction of the structure model

The initial models of the COF structures were built in BIOVIA Materials Studio 2017 (17.1.0.48) © 2016 Dassault Systèmes using Forcite module geometry optimizations with universal force fields and Ewald electrostatic and van der Waals summation methods.

X-ray powder diffraction

X-ray powder diffraction patterns were collected at room temperature on a STOE Stadi-P diffractometer (Co $K_{\alpha}1$, λ = 1.79 Å), Ge(111) Johann monochromator, Mythen 1K detector with an opening angle of 19 ° in Debye-Scherrer geometry. The samples were measured inside sealed glass capillaries (ϕ 0.7 mm capillary). For improved particle statistics the samples were spun. The samples were measured from 2–35 ° 2θ over 3x3 h.

Rietveld refinements

Rietveld refinements were performed using TOPAS-Academic v6.[5] The instrumental profile was fit to a measurement of NIST 640d Si standard using a TCHZ peak shape with full axial and zero-error corrections, which were fixed for subsequent refinements. **HT** and **LT** were fit using additional corrections for Gaussian and Lorentzian crystallite size and strain broadening and lattice parameters associated with different models used. An isotropic atomic displacement parameters B_{iso} = 5.0 Å^2 was fixed. For non-eclipsed models, the translation of the second layer in the unit cell was allowed to refine in the *xy* plane.

Synchrotron measurements

Total scattering measurements were carried out using the high energy x-ray beamline ID-31 at the European Synchrotron Radiation Facility (ESRF). The rapid acquisition PDF method (RAPDF)[6] was used with a Pilatus3 X CdTe 2M detector from Dectris (1475 × 1679 pixels, 172 × 172 µm^2 each) and sample-to-detector distances of both 773.4 mm and 273.5 mm. Samples were loaded into 1.5 mm ID polyimide capillaries and measured at ambient conditions. The incident energy of the X-rays was 68.54 keV (λ = 0.1809 Å). Calibration of the experimental setup was performed with a NIST 674b CeO_2 standard dataset, collected at room temperature, using the pyFAI software.[7] Further mask generation, polarization correction, and azimuthal integration of the 2D patterns was performed using the xpdtools software suite.[8,9]

Duplicate measurements were performed using beamline I15-1 at Diamond Light source, Harwell Science and Innovation Campus, Oxfordshire, UK. The measurements were also performed using RAPDF mode with a Perkin Elmer XRD 1611 CP3 detector (4096 × 4096 pixels, 100 × 100 µm^2 each) and an incident energy of approximately 40. keV (λ =0.31 Å). Samples were loaded into 1.5 mm ID VITREX borosilicate capillaries and measured at ambient conditions. Calibration of the experimental setup was performed with a measurement of Si SRM640c standard, collected at room temperature, using DAWN software.[10]

Pair distribution function (PDF) analysis

Total scattering measurements over a wide range of momentum transfer and with good statistics are required to obtain suitable PDFs for structure analysis. The coherent diffraction intensities are normalized to obtain the total scattering structure function $S(Q)$, which is then Fourier transformed by

$$G(r) = \frac{2}{\pi}\int_{Q_{min}}^{Q_{max}} Q[S(Q)-1]sin(Qr)dQ. \qquad \text{Equation SI-1}$$

$F(Q) = Q[S(Q)-1]$ is the reduced total scattering structure function, and $G(r)$ is the PDF.[11,12] In practice, values of Q_{min} and Q_{max} are determined by the experimental setup, and Q_{max} is often reduced below the experimental maximum to reduce the effects of low signal-to-noise in the high-Q region on the Fourier transformation. PDF data was obtained using the data normalization and correction procedures implemented in PDFgetX3 and xPDFsuite.[13–16]

The PDF gives the scaled probability of finding two atoms in a material a distance r apart and is relative to the density of atom pairs in the material. For a macroscopic scatterer, $G(r)$ is calculated from a known structure model according to

$$G(r) = 4\pi r[\rho(r) - \rho_0], \qquad \text{Equation SI-2}$$

$$\rho(r) = \frac{1}{4\pi r^2 N}\sum_i \sum_{j\neq i} \frac{f_i f_j}{\langle f\rangle^2}\delta(r - r_{ij}). \qquad \text{Equation SI-3}$$

Here, ρ_0 is the average number density of the material and $\rho(r)$ is the local atomic pair density, which is the mean weighted density of neighbor atoms at distance r from an atom at the origin. The sums in $\rho(r)$ run over all atoms in the sample, f_i is the scattering factor of atom i, $\langle f\rangle$ is the average scattering factor and r_{ij} is the distance between atoms i and j. In this study, Equations SI-2 and SI-3 were used to fit the PDF generated from a structure model to the experimental PDFs in using the program PDFgui.[17] The delta functions in Equation SI-3 were Gaussian-broadened. . PDF modeling was performed by adjusting the lattice parameters, anisotropic atomic displacement parameters (ADPs) where U11=U22≠U33, correlated motion of neighboring atoms using δ2 and sratio (rcut = 5.1) to sharpen intramolecular correlations, domain size (spdiameter), a global scale factor, and phase-specific scale factors for multiphase fits. The resolution parameters due to Q-space resolution, Q_{damp} = 0.00733 Å^{-1}, and due to variable Q-resolution and high-Q noise, Q_{broad} = 0.00656 Å^{-1}, were determined from CeO_2 and fixed for further fitting. The refinements were run by minimizing R_{wp}, for the set of refined parameters P in the model, calculated as

$$R_{wp} = \sqrt{\left(\sum_{i=1}^{n}[G_{obs}(r_i) - G_{calc}(r_i,P)]^2\right)/\left(\sum_{i=1}^{n} G_{obs}(r_i)^2\right)}. \qquad \text{Equation SI-4}$$

NMR and IR data

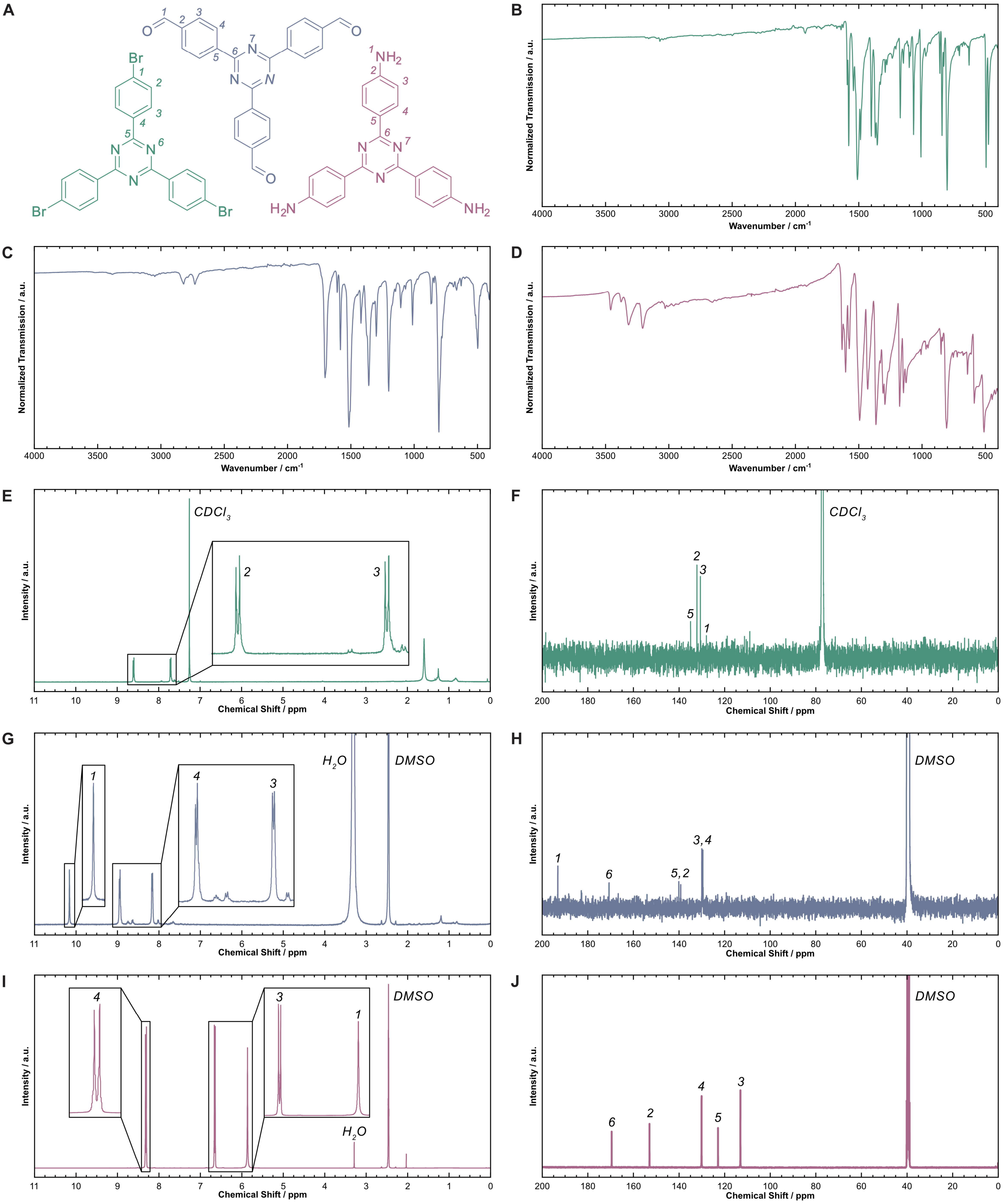

Figure SI-1 (A) Structural formulas of 2,4,6-tris(4-bromophenyl)-1,3,5-triazine , 4,4',4''-(1,3,5-triazine-2,4,6-triyl)tribenzaldehyde, and 2,4,6-tris(4-aminophenyl)-1,3,5-triazine with atom labels. (B,C,D) Infrared spectra of these molecules. (E,G,I) ^{1}H NMR spectra of these molecules with assignments. (F,H,J) ^{13}C NMR spectra of these molecules with assignments.

Electron microscopy

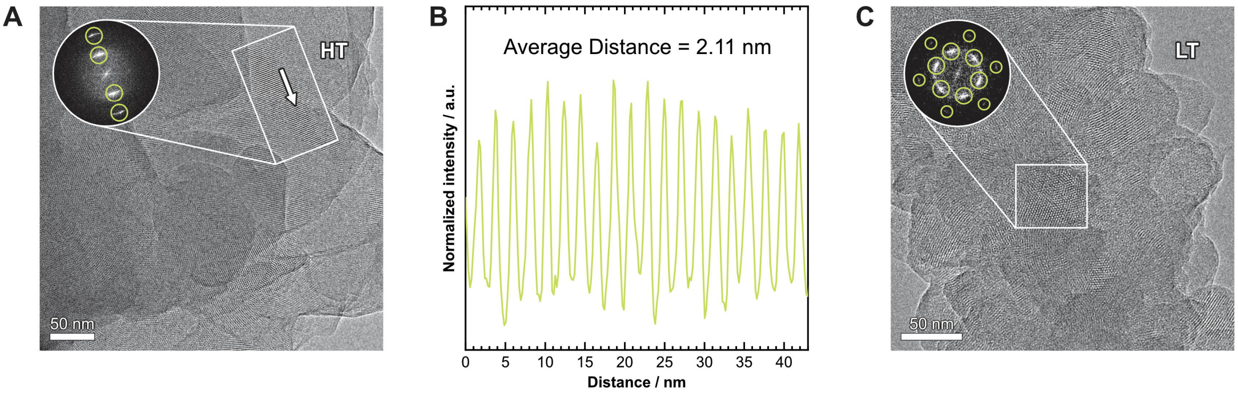

Figure SI-2 (A) Transmission electron micrograph of **HT**, with inset fast Fourier transform of the highlighted area, which depicts pore channels as viewed from the side. Intensity maxima correspond to the 100 and 200 planes, with 2.1 nm and 1.1 nm, respectively. (B) Intensity profile along the arrow. (C) Transmission electron micrograph of **LT**, with inset fast Fourier transform of the highlighted area, which depicts pores as viewed from above. Intensity maxima correspond to the 100 and 110 planes, with 2.0 nm and 1.1 nm, respectively. Sample contraction due to degradation from beam damage is generally more pronounced in disordered samples with smaller, less stable crystallites, such as **LT**.

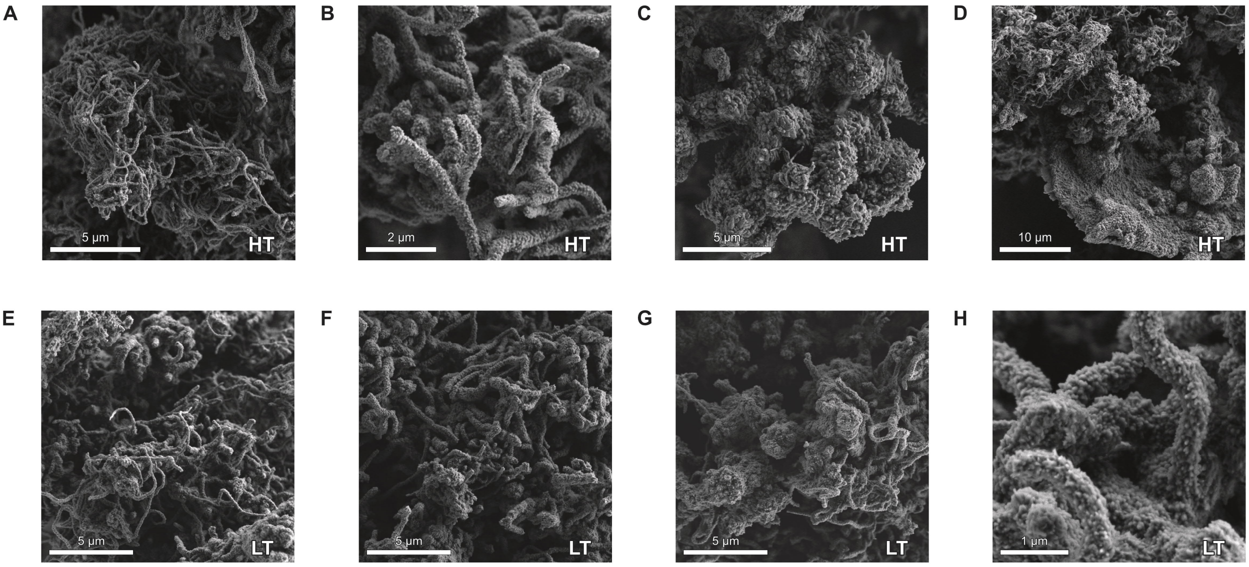

Figure SI-3 (A-D) Additional scanning electron micrographs of **HT** and (E-H) of **LT**.

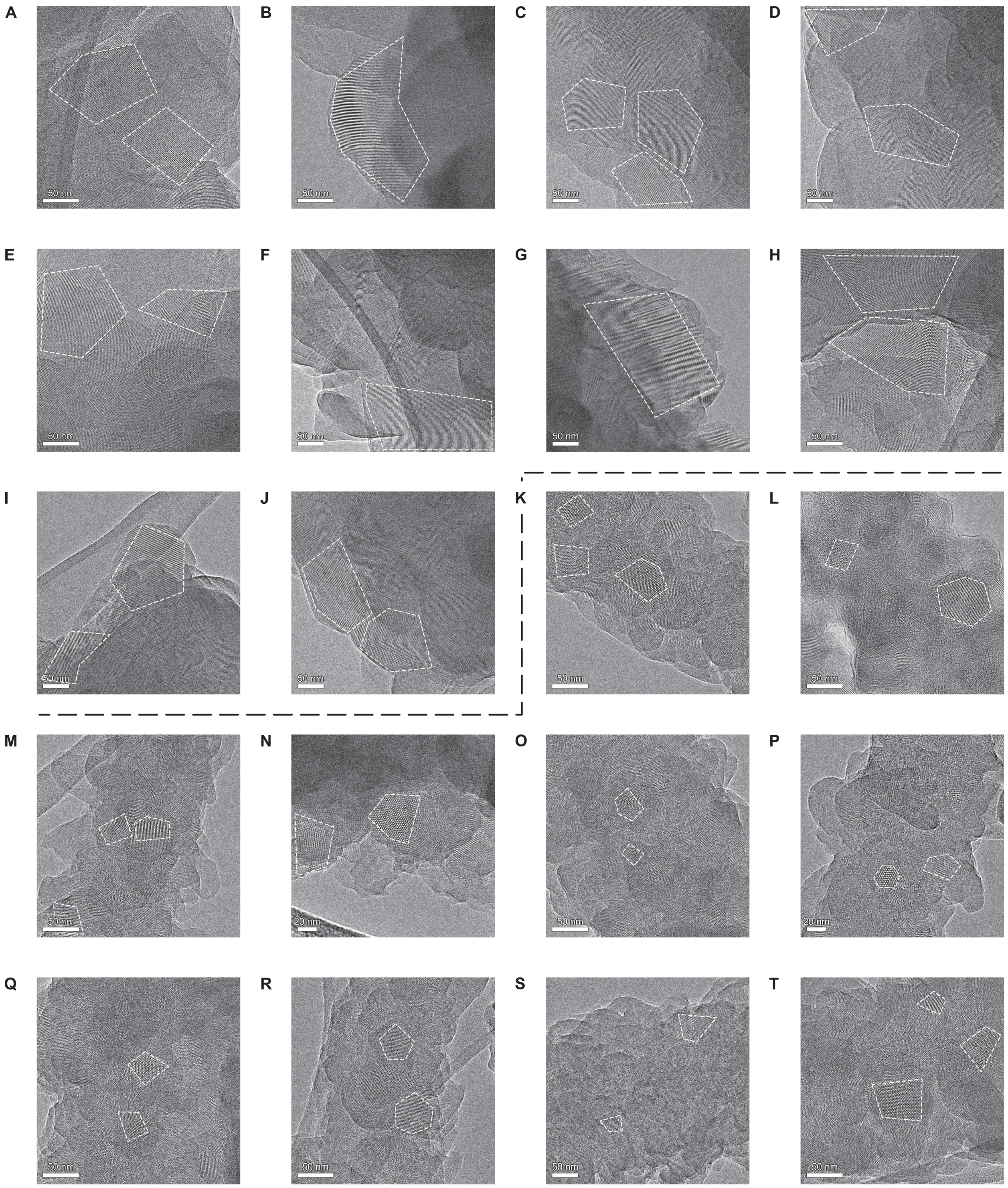

Figure SI-4 (A–J) Additional transmission electron micrographs of **HT** and (K–T) of **LT**, with some crystallites highlighted. These examples illustrate the significant difference in average crystallite sizes between the two samples. Morphology differences are also visible, in that **HT** forms noticeably bigger aggregates.

Physisorption analysis

$$\frac{p}{\boldsymbol{n}(p^0 - p)} = \frac{p/p^0}{\boldsymbol{n}(1 - p/p^0)} = \frac{1}{\boldsymbol{n}_\mathrm{m} C} + \frac{C - 1}{\boldsymbol{n}_\mathrm{m} C}(p/p^0)$$

Equation SI-5 Brunauer-Emmett-Teller (BET) equation, with equilibrium pressure p, saturation pressure p^0, specific amount absorbed $\boldsymbol{n}$, specific monolayer capacity $\boldsymbol{n}_\mathrm{m}$, and constant C, which constitutes a measure of the monolayer absorption energy.[3,18] The linear section of the BET plot, $(p/p^0)[\boldsymbol{n}(1\text{-}p/p^0)]$ over p/p^0, has the slope $(C\text{-}1)/(\boldsymbol{n}_\mathrm{m}C)$ and the intercept $1/(\boldsymbol{n}_\mathrm{m}C)$.

$$\frac{1}{S + I} = \left(\frac{C - 1}{\boldsymbol{n}_\mathrm{m} C} + \frac{1}{\boldsymbol{n}_\mathrm{m} C}\right)^{-1} = \left(\frac{C}{\boldsymbol{n}_\mathrm{m} C}\right)^{-1} = \boldsymbol{n}_\mathrm{m}$$

Equation SI-6 Calculation of the specific monolayer capacity from the slope S and intercept I of the linear fit of the BET plot.[18]

$$1 + \frac{S}{I} = 1 + \left(\frac{C - 1}{\boldsymbol{n}_\mathrm{m} C}\right)\left(\frac{1}{\boldsymbol{n}_\mathrm{m} C}\right)^{-1} = 1 + \frac{(C - 1)(\boldsymbol{n}_\mathrm{m} C)}{\boldsymbol{n}_\mathrm{m} C} = C$$

Equation SI-7 Calculation of C from the slope S and intercept I of the linear fit of the BET plot.[18]

$$a_\mathrm{S}(\mathrm{BET}) = \boldsymbol{n}_\mathrm{m} \times L \times \sigma_\mathrm{m} = \boldsymbol{n}_\mathrm{m} \times 6.02214076 \times 10^{23} \frac{1}{\mathrm{mol}} \times 0.142\ \mathrm{nm}^2$$

Equation SI-8 The BET area/apparent surface area a_s(BET) in $m^2\ g^{-1}$ is equal to the product of the specific monolayer capacity $\boldsymbol{n}_\mathrm{m}$ in mol g^{-1}, Avogadro constant L, and the molecular cross-sectional area σ_m of the adsorbate (here, argon).[3]

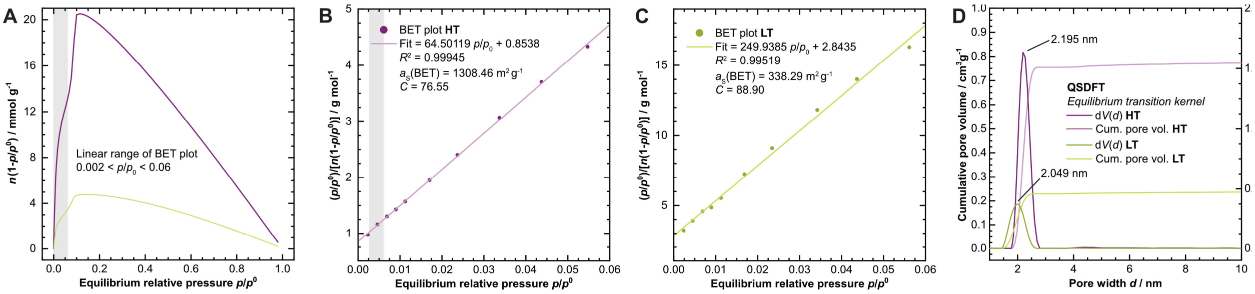

Figure SI-5 (A) The BET equation (Equation SI-5) should only be applied where the term $\boldsymbol{n}(1\text{-}p/p^0)$ continuously increases with p/p^0, as shown for **HT** (purple) and **LT** (green). The sections corresponding to the linear ranges of the BET plots are highlighted. (B) Linear range of the BET plot of **HT** and (C) of **LT** with linear fit and calculated values thereof. (D) Cumulative pore volumes and PSDs for **HT** and **LT** as calculated by QSDFT using the equilibrium transition kernel (for comparison see Figure 3B and D). Note the deviating pore size for **LT**. Limited diffusion of the adsorbate causes a hysteresis loop and the PSD derived from the desorption branch becomes inaccurate. We observed artifacts in the form of cumulative pore volume at slightly higher pore widths in both materials, which only occur with the adsorption branch kernel.[19]

Rietveld refinements

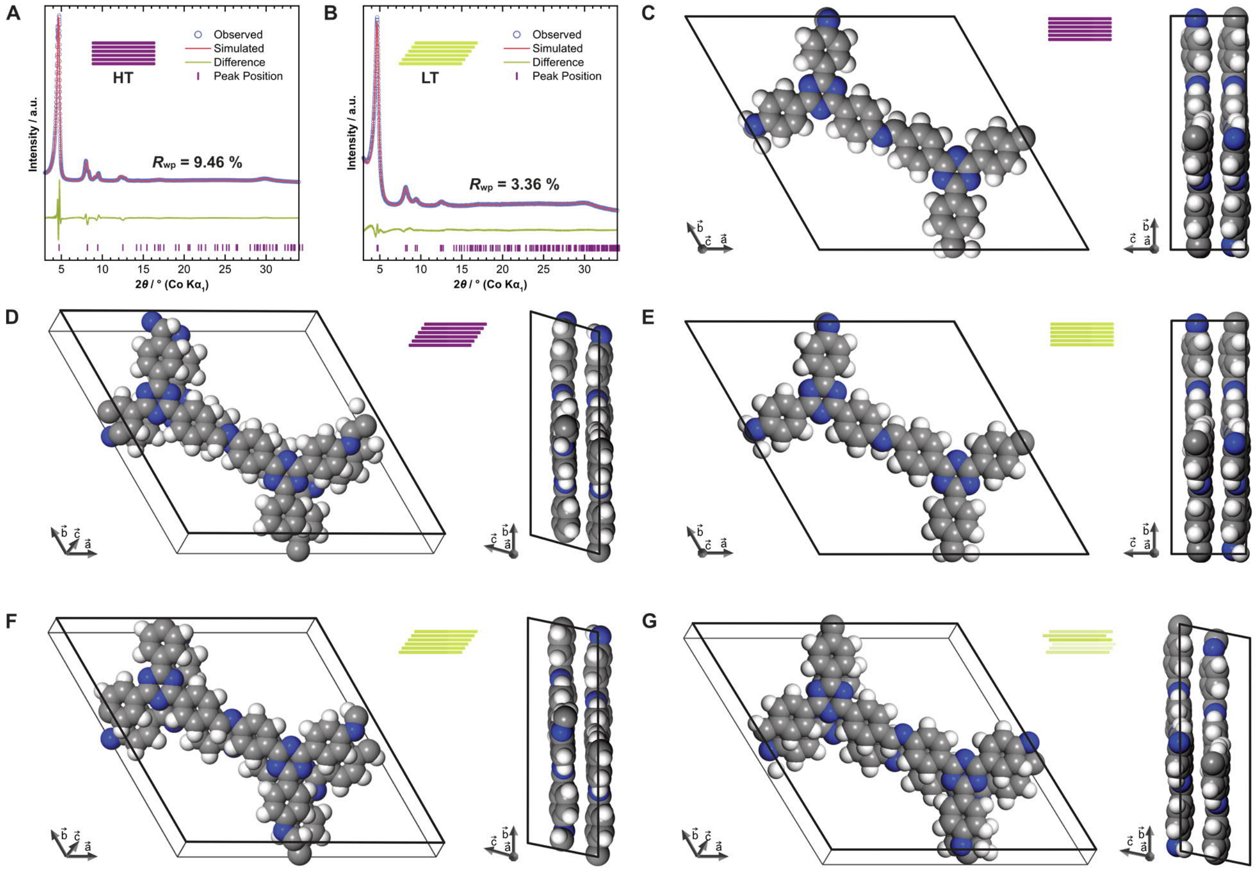

Figure SI-6 (A) Diffraction patterns of HT with best obtained fit by Rietveld refinement assuming eclipsed stacking and (B) of LT assuming unidirectionally slipped stacking. (C) View onto and across unit cells obtained from Rietveld fits of HT assuming eclipsed and (D) unidirectionally slipped stacking. (E) View onto and across unit cells obtained from Rietveld fits of LT assuming eclipsed, (F) unidirectionally slipped, and (G) random stacking. The quality of these Rietveld fits was not affected significantly by the imine orientation, thus only antiparallel orientation of the imine groups was assumed, based on previous evidence.[20] For refinements with random stacking, an optimized slip stacked unit cell was used to construct supercells containing 200 layers each using appropriate stacking vectors.

Reduced total scattering structure functions

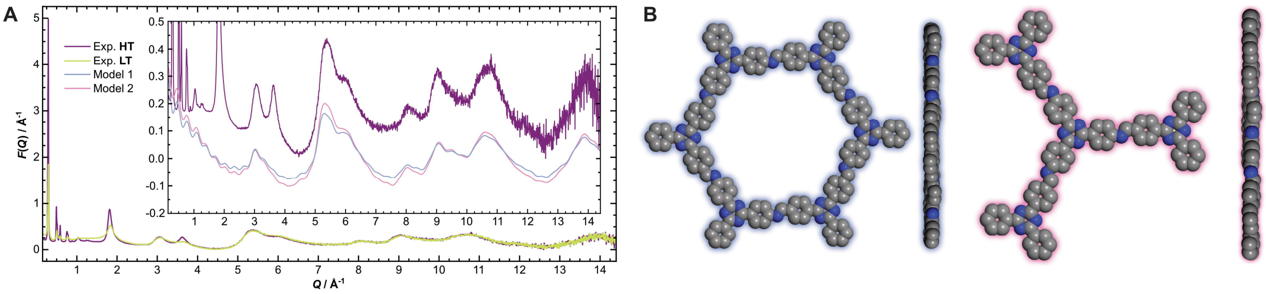

Figure SI-7 (A) Comparison of reduced total scattering structure functions F(Q) of HT (purple) and LT (green) collected using synchrotron radiation (ESRF) over the same Q-space used to derive the pair distribution functions G(r). Inset: simulated reduced total scattering structure functions for two single-layer structure models compared to HT.[21] (B) Views onto and across the structures of model 1 and 2.

Pair distribution function analysis

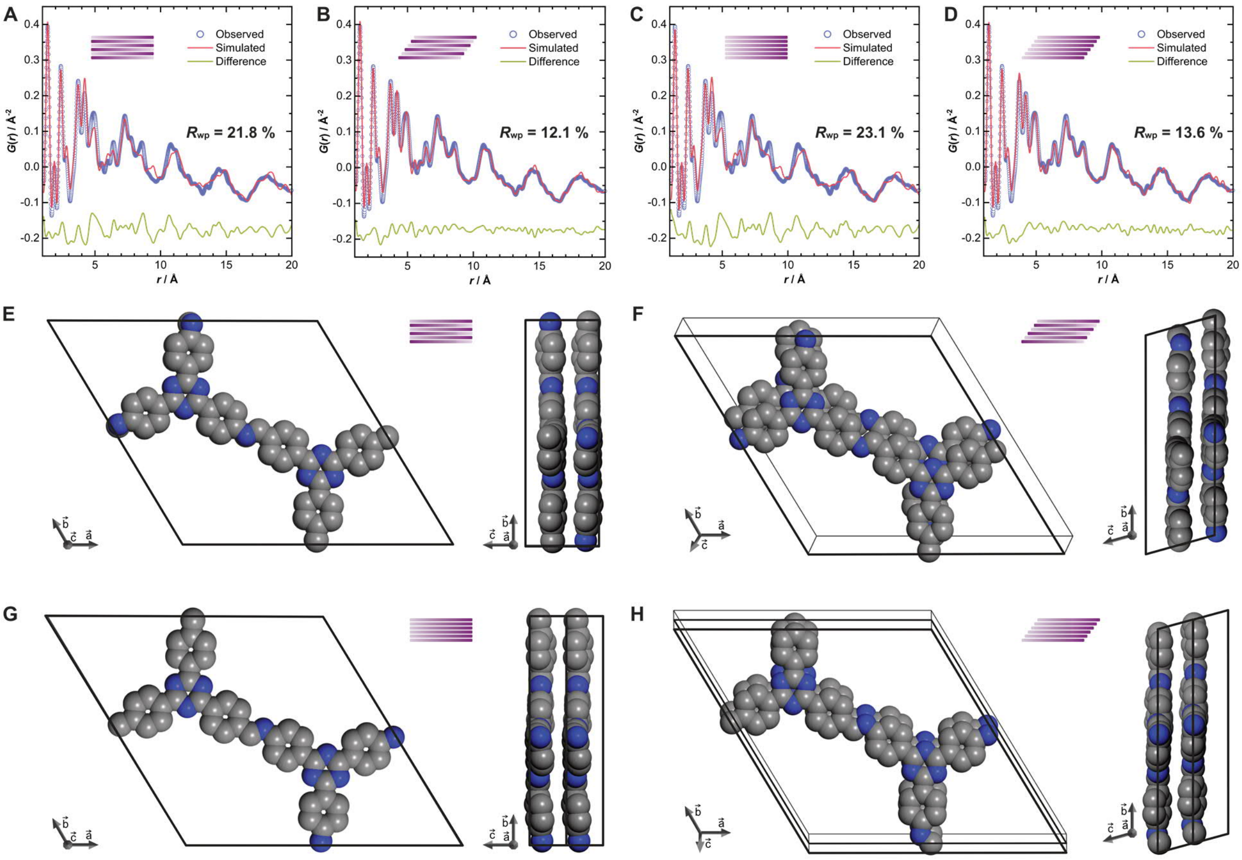

Figure SI-8 (A) Best obtained PDF fits over 1 Å–20 Å from structure refinements of HT, assuming antiparallel eclipsed, (B) antiparallel unidirectionally slipped, (C) parallel eclipsed, and (D) parallel unidirectionally slipped stacking. (E) View onto and across unit cells obtained from PDF fits of HT assuming antiparallel eclipsed, (F) antiparallel unidirectionally slipped, (G) parallel eclipsed, and (H) parallel unidirectionally slipped stacking.

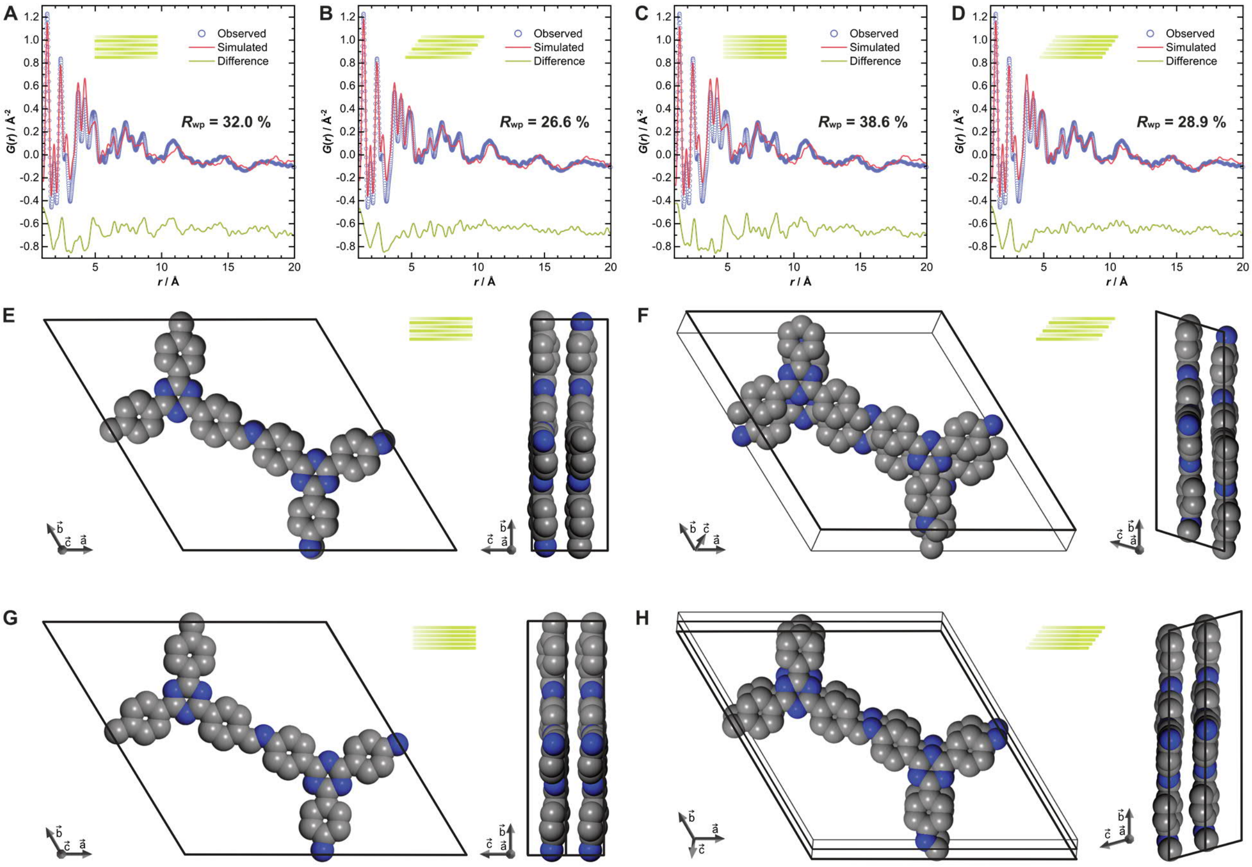

Figure SI-9 (A) Best obtained PDF fits over 1 Å–20 Å from structure refinements of LT, assuming antiparallel eclipsed, (B) antiparallel unidirectionally slipped, (C) parallel eclipsed, and (D) parallel unidirectionally slipped stacking. (E) View onto and across unit cells obtained from PDF fits of LT assuming antiparallel eclipsed, (F) antiparallel unidirectionally slipped, (G) parallel eclipsed, and (H) parallel unidirectionally slipped stacking.

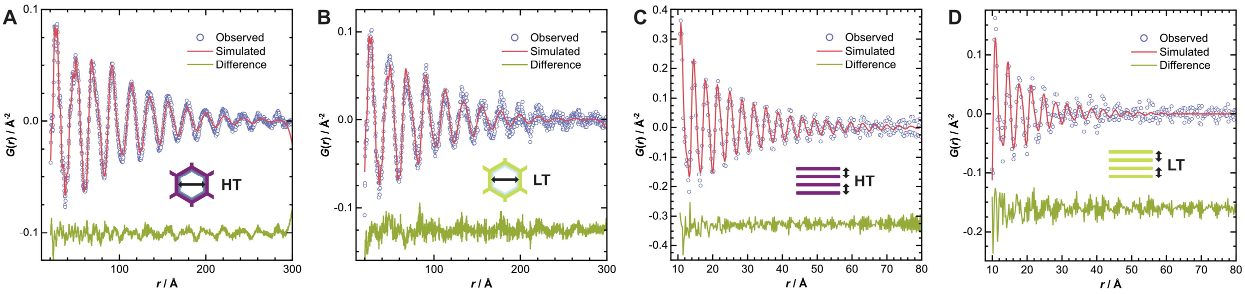

Figure SI-10 (A) Long-range pair distribution function analysis of HT and (B) of LT. The experimental data is fit using a simplified structure model, i.e., a hexagonal unit cell with one carbon atom at the origin. The coherence length here is also much smaller for LT, indicating higher disorder and smaller crystallites. (C) Pair distribution function analysis of HT, and (D) of LT, where the stacking component is isolated by truncating the total scattering structure function F(Q) to Q values above 1.5 Å^{-1}. The experimental data is fit using a simplified structure model, i.e., a hexagonal graphite unit cell. The fit is better and the coherence length is higher for HT than for LT, which indicates significantly higher long-range order in the stacking direction.

Rietveld and PDF stacking fault simulations

A model of the faultless structure of the TTI-COF could be obtained from HT. The unit cell contains two layers with the orientation of each layer flipped horizontally with respect to the preceding one, which leads to an overall ABAB-type stacking order. The layers are stacked in a slipped confirmation with the center of each C3N6 ring being shifted towards the carbon atom of the preceding layer. Two types of layer shifts can be observed: Layer-type B (Figure SI-11A, blue glow effect) is shifted (stacking vector S1-1) with respect to the preceding layer-type A (yellow glow effect) towards a carbon atom of the triazine. The layer-type A is shifted (stacking vector S2-1) with respect to the preceding layer-type B towards a carbon atom of the benzene ring next to the triazine (Figure SI-11B). Due to the trigonal molecular setup of the layers, different orientations of the stacking vectors lead to equally favorable stacking patterns. Thus faulting scenarios similar to stacking fault disorder observed in K-PHI and H-PHI can be derived.[22]

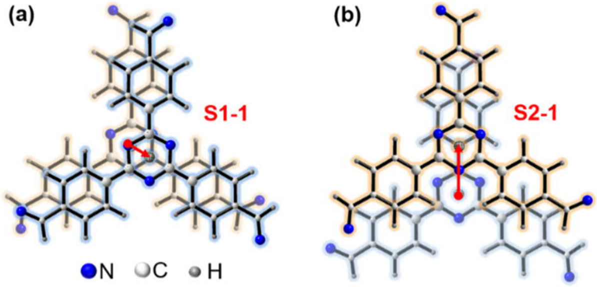

Figure SI-11 Stacking order in TTI-COF, the layers are stacked in an ABAB fashion, A-type layers are highlighted with a yellow and B-type layers with a blue glow effect. The stacking vectors are represented by red arrows.

Faulting Scenario 1

The trigonal setup of the layers suggests that the orientation of the stacking vectors can vary. Both the short stacking vector S1-1 (Figure SI-12A-C) and the long stacking vector S2-1 (Figure SI-12D-F) can be twisted by 120 ° and 240 °, which leads to two alternative stacking vectors both for S1-1 and for S2-1. For this faulting scenario it was assumed that the ABAB like stacking order is always maintained, that transitions from layer-type A to layer-type B always occur by a short stacking vector (S1-1, S1-2 or S1-3) and transitions from layer-type B to layer-type A by a long stacking vector (S2-1, S2-2, S2-3) and that all possible stacking orders are equally favorable. This led to a 6x6 transition probability matrix (Table SI-1) with all transition probabilities constrained by the global fault probability P_f.

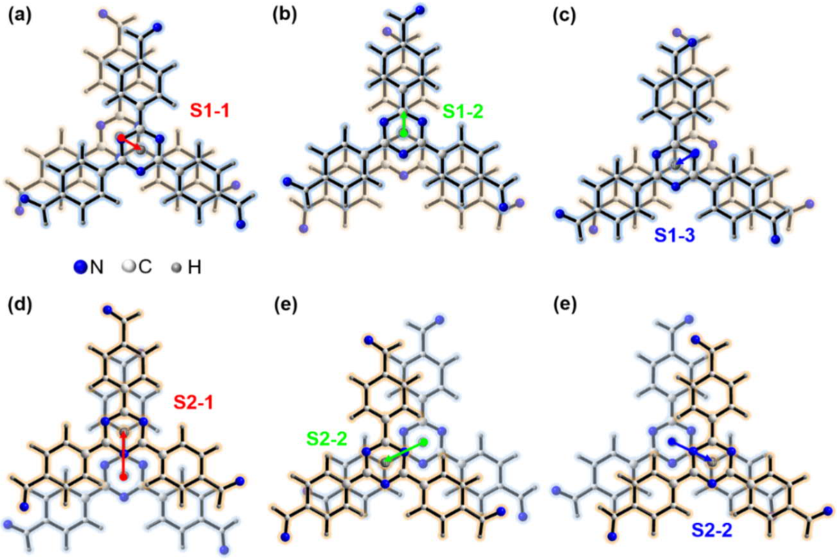

Figure SI-12 Illustration of faulting scenario 1. A-type layers are highlighted with a yellow and B-type layers with a blue glow effect. The stacking vectors are represented by red, green and blue arrows.

Table SI-1 Transition probability matrix used to describe faulting scenario 1. The applied stacking vectors are in in bold font. By assuming that all alternative stacking orders are equally favorable, that fault probabilities were constrained to one global parameter: P_f, with P_f = 0 representing the faultless structure of TTI-COF.

	Transition from↓/to→	Layer-type A S1-1	S1-2	S1-3	Layer-type B S2-1	S2-2	S2-3
Layer-type A	S1-1	0	0	0	$1-P_f$<br>S1-1	$P_f/2$<br>S1-2	$P_f/2$<br>S1-3
	S1-2	0	0	0	$P_f/2$<br>S1-1	$1-P_f$<br>S1-2	$P_f/2$<br>S1-3
	S1-3	0	0	0	$P_f/2$<br>S1-1	$P_f/2$<br>S1-2	$1-P_f$<br>S1-3
Layer-type B	S2-1	$1-P_f$<br>S2-1	$P_f/2$<br>S2-2	$P_f/2$<br>S2-3	0	0	0
	S2-2	$P_f/2$<br>S2-1	$1-P_f$<br>S2-2	$P_f/2$<br>S2-3	0	0	0
	S2-3	$P_f/2$<br>S2-1	$P_f/2$<br>S2-2	$1-P_f$<br>S2-3	0	0	0

Faulting Scenario 2

For faulting scenario 2 only one orientation of the stacking vectors was used, but the transitions between the layer-types were allowed to occur both by a short (S1-1) and a long (S2-1) stacking vector (Figure SI-13). The 2x2 transition probability matrix describing this faulting scenario is given in Table SI-2. It was assumed that faults in the transition from layer-type A to layer-type B have the same probability as faults in the transition from layer-type B to layer-type A, which led to the global fault probability P_f.

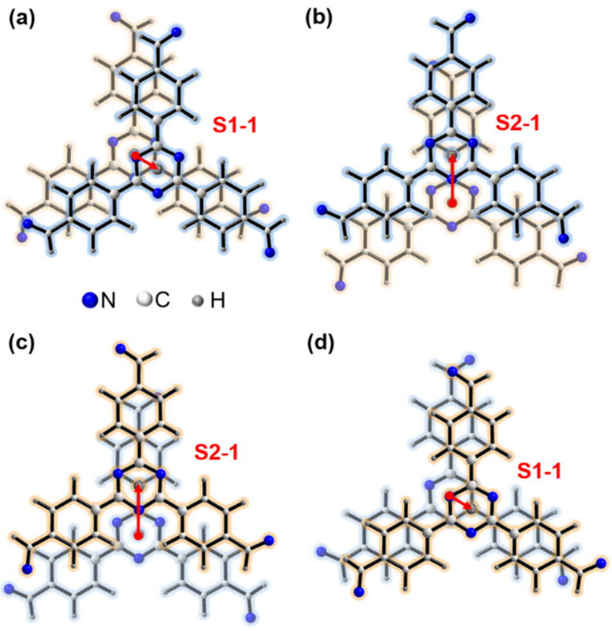

Figure SI-13 Illustration of faulting scenario 2. A-type layers are highlighted with a yellow and B-type layers with a blue glow effect. The stacking vectors are represented by red, green and blue arrows.

Table SI-2 Transition probability matrix used to describe faulting scenario 2. By assuming that all alternative stacking orders are equally favorable, that fault probabilities were constrained to one global parameter: P_f, with P_f = 0 representing the faultless structure of TTI-COF.

	Transition	Layer-type A		Layer-type B	
	from↓/to→	S1-1	S2-1	S2-1	S1-1
Layer-type A	S1-1	0	0	1-P_f<br>S1-1	P_f<br>S2-1
	S2-1	0	0	P_f<br>S1-1	1-P_f<br>S2-1
Layer-type B	S2-1	1-P_f<br>S2-1	P_f<br>S1-1	0	0
	S1-1	P_f<br>S2-1	1-P_f<br>S1-1	0	0

Faulting Scenario 3

Faulting scenario 3 is a combination of scenarios 1 and 2. It was assumed that the ABAB stacking order is still maintained, but the transition from one layer-type to the other can occur both via the short and the long stacking vector with all possible orientations (Figure SI-14). The corresponding 12x12 transition probability matrix is given in Table SI-3. All faulting probabilities were constrained to one parameter, P_f, describing the global fault probability as it was assumed that all possible stacking orders are equally favorable.

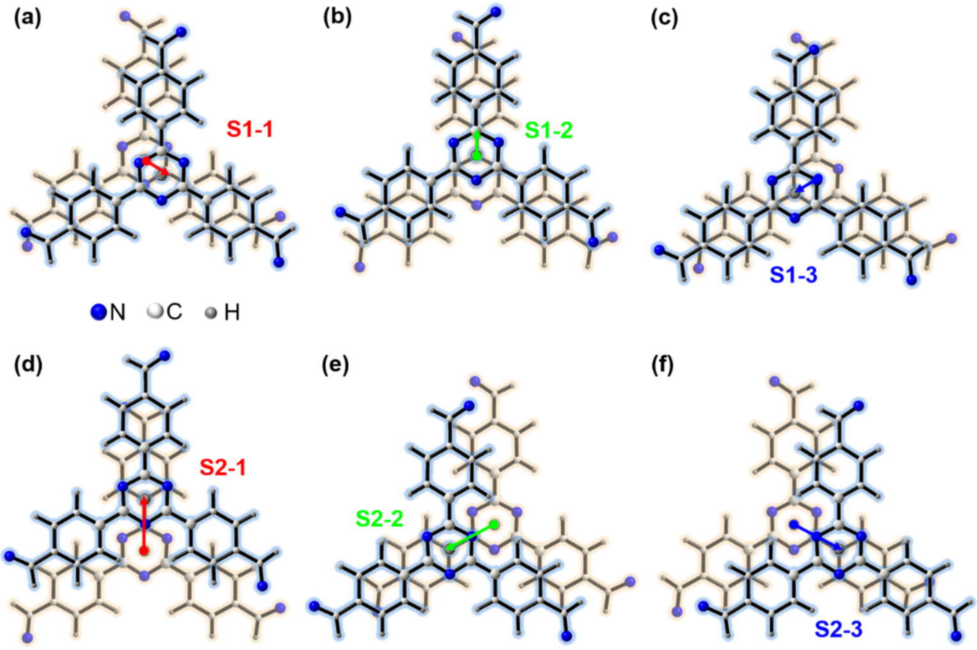

Figure SI-14 Illustration of faulting scenario 3. A-type layers are highlighted with a yellow and B-type layers with a blue glow effect. The stacking vectors are represented by red, green and blue arrows.

Table SI-3 Transition probability matrix used to describe faulting scenario 3. By assuming that all alternative stacking orders are equally favorable, that fault probabilities were constrained to one global parameter: P_f, with P_f = 0 representing the faultless structure of TTI-COF.

	Transition	Layer-type A						Layer-type B					
	from↓/to→	S1-1	S1-2	S1-3	S2-1	S2-2	S2-3	S2-1	S2-2	S2-3	S1-1	S1-2	S1-3
Layer-type A	S1-1	0	0	0	0	0	0	1-P_f<br>S1-1	P_f/5<br>S1-2	P_f/5<br>S1-3	P_f/5<br>S2-1	P_f/5<br>S2-2	P_f/5<br>S2-3
	S1-2	0	0	0	0	0	0	P_f/5<br>S1-1	1-P_f<br>S1-2	P_f/5<br>S1-3	P_f/5<br>S2-1	P_f/5<br>S2-2	P_f/5<br>S2-3
	S1-3	0	0	0	0	0	0	P_f/5<br>S1-1	P_f/5<br>S1-2	1-P_f<br>S1-3	P_f/5<br>S2-1	P_f/5<br>S2-2	P_f/5<br>S2-3
	S2-1	0	0	0	0	0	0	P_f/5<br>S1-1	P_f/5<br>S1-2	P_f/5<br>S1-3	1-P_f<br>S2-1	P_f/5<br>S2-2	P_f/5<br>S2-3
	S2-2	0	0	0	0	0	0	P_f/5<br>S1-1	P_f/5<br>S1-2	P_f/5<br>S1-3	P_f/5<br>S2-1	1-P_f<br>S2-2	P_f/5<br>S2-3
	S2-3	0	0	0	0	0	0	P_f/5<br>S1-1	P_f/5<br>S1-2	P_f/5<br>S1-3	P_f/5<br>S2-1	P_f/5<br>S2-2	1-P_f<br>S2-3
Layer-type B	S2-1	1-P_f<br>S2-1	P_f/5<br>S2-2	P_f/5<br>S2-3	P_f/5<br>S1-1	P_f/5<br>S1-2	P_f/5<br>S1-3	0	0	0	0	0	0
	S2-2	P_f/5<br>S2-1	1-P_f<br>S2-2	P_f/5<br>S2-3	P_f/5<br>S1-1	P_f/5<br>S1-2	P_f/5<br>S1-3	0	0	0	0	0	0

S2-3	$P_f/5$<br>S2-1	$P_f/5$<br>S2-2	$1-P_f$<br>S2-3	$P_f/5$<br>S1-1	$P_f/5$<br>S1-2	$P_f/5$<br>S1-3	0	0	0	0	0	0
S1-1	$P_f/5$<br>S2-1	$P_f/5$<br>S2-2	$P_f/5$<br>S2-3	$1-P_f$<br>S1-1	$P_f/5$<br>S1-2	$P_f/5$<br>S1-3	0	0	0	0	0	0
S1-2	$P_f/5$<br>S2-1	$P_f/5$<br>S2-2	$P_f/5$<br>S2-3	$P_f/5$<br>S1-1	$1-P_f$<br>S1-2	$P_f/5$<br>S1-3	0	0	0	0	0	0
S1-3	$P_f/5$<br>S2-1	$P_f/5$<br>S2-2	$P_f/5$<br>S2-3	$P_f/5$<br>S1-1	$P_f/5$<br>S1-2	$1-P_f$<br>S1-3	0	0	0	0	0	0

Faulting Scenario 4

In faulting scenario 4 faults within the ABAB-type stacking order were considered. Transitions from layer-type A to layer type B were assumed to occur via the short stacking vector S1-1, whereas transitions within the same layer type were assumed to occur only via the long stacking vector S2-1 (Figure SI-15). The transition probability matrix is presented in Table SI-4.

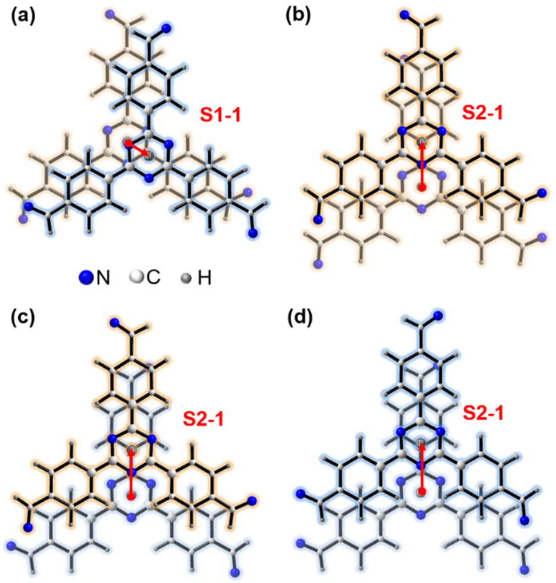

Figure SI-15 Illustration of faulting scenario 4. A-type layers are highlighted with a yellow and B-type layers with a blue glow effect. The stacking vectors are represented by red, green and blue arrows.

Table SI-4 Transition probability matrix used to describe faulting scenario 4. By assuming that all alternative stacking orders are equally favorable, that fault probabilities were constrained to one global parameter: P_f, with $P_f = 0$ representing the faultless structure of TTI-COF.

	Transition	Layer-type A		Layer-type B
	from↓/to→	S1-1	S2-1	S2-1
Layer-type A	S1-1	0	P_f<br>S2-1	1-P_f<br>S1-1
	S2-1	0	P_f<br>S2-1	1-P_f<br>S1-1
Layer-type B	S2-1	1-P_f<br>S2-1	0	P_f<br>S2-1

Faulting Scenario 5

Faulting scenario 5 is a combination of scenario 3 and 4 (Figure SI-16). The transition probability matrix is shown in Table SI-5.

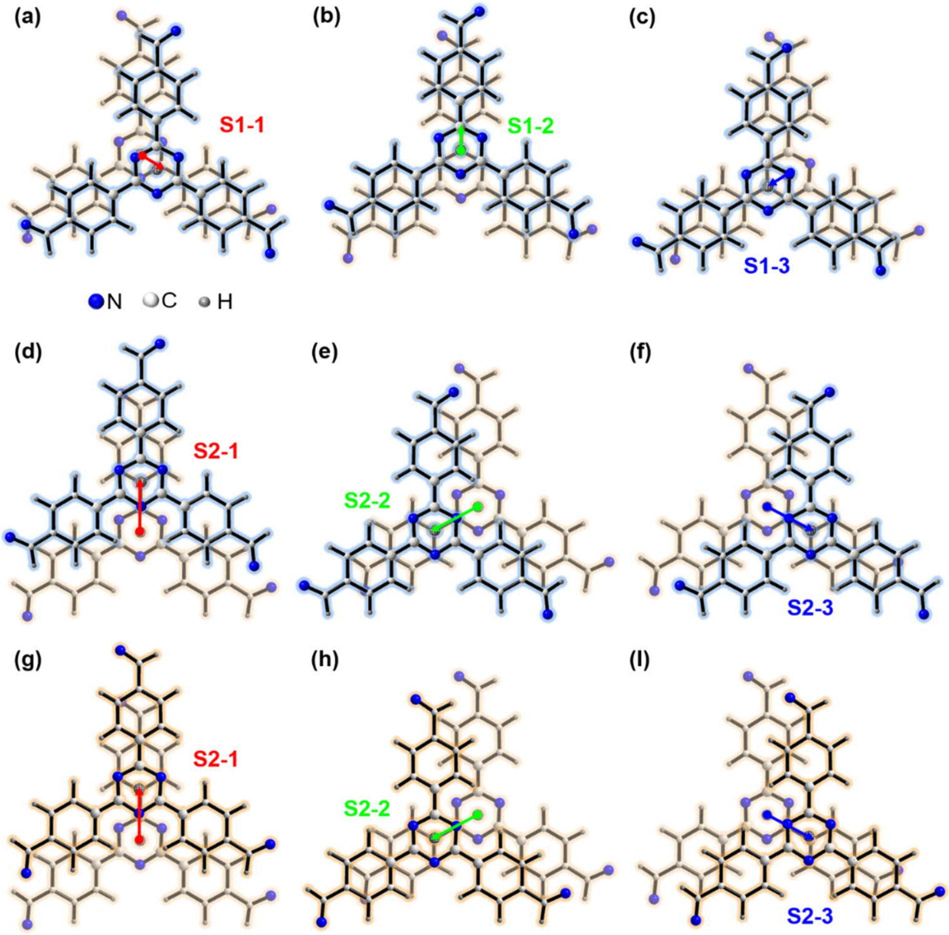

Figure SI-16 Illustration of faulting scenario 5. A-type layers are highlighted with a yellow and B-type layers with a blue glow effect. The stacking vectors are represented by red, green and blue arrows.

Table SI-5 Transition probability matrix used to describe faulting scenario 5. Two parameters were used to describe the faulting scenario. P_f describes faults in the layer orientation and P_x describes faults in the overall ABAB-type layer stacking.

	Transition	Layer-type A						Layer-type B					
	from↓/to→	S1-1	S1-2	S1-3	S2-1	S2-2	S2-3	S2-1	S2-2	S2-3	S1-1	S1-2	S1-3
	S1-1	0	0	0	$P_x/3$ S2-1	$P_x/3$ S2-2	$P_x/3$ S2-3	$1-P_f-P_x/6$ S1-1	$P_f/5-P_x/6$ S1-2	$P_f/5-P_x/6$ S1-3	$P_f/5-P_x/6$ S2-1	$P_f/5-P_x/6$ S2-2	$P_f/5-P_x/6$ S2-3
	S1-2	0	0	0	$P_x/3$ S2-1	$P_x/3$ S2-2	$P_x/3$ S2-3	$P_f/5-P_x/6$ S1-1	$1-P_f-P_x/6$ S1-2	$P_f/5-P_x/6$ S1-3	$P_f/5-P_x/6$ S2-1	$P_f/5-P_x/6$ S2-2	$P_f/5-P_x/6$ S2-3
	S1-3	0	0	0	$P_x/3$ S2-1	$P_x/3$ S2-2	$P_x/3$ S2-3	$P_f/5-P_x/6$ S1-1	$P_f/5-P_x/6$ S1-2	$1-P_f-P_x/6$ S1-3	$P_f/5-P_x/6$ S2-1	$P_f/5-P_x/6$ S2-2	$P_f/5-P_x/6$ S2-3
Layer-type A	S2-1	0	0	0	$P_x/3$ S2-1	$P_x/3$ S2-2	$P_x/3$ S2-3	$P_f/5-P_x/6$ S1-1	$P_f/5-P_x/6$ S1-2	$P_f/5-P_x/6$ S1-3	$1-P_f-P_x/6$ S2-1	$P_f/5-P_x/6$ S2-2	$P_f/5-P_x/6$ S2-3
	S2-2	0	0	0	$P_x/3$ S2-1	$P_x/3$ S2-2	$P_x/3$ S2-3	$P_f/5-P_x/6$ S1-1	$P_f/5-P_x/6$ S1-2	$P_f/5-P_x/6$ S1-3	$P_f/5-P_x/6$ S2-1	$1-P_f-P_x/6$ S2-2	$P_f/5-P_x/6$ S2-3
	S2-3	0	0	0	$P_x/3$ S2-1	$P_x/3$ S2-2	$P_x/3$ S2-3	$P_f/5-P_x/6$	$P_f/5-P_x/6$	$P_f/5-P_x/6$	$P_f/5-P_x/6$	$P_f/5-P_x/6$	$1-P_f-P_x/6$

								S1-1	S1-2	S1-3	S2-1	S2-2	S2-3
Layer-type B	S2-1	$1-P_f-P_x/6$<br>S2-1	$P_f/5-P_x/6$<br>S2-2	$P_f/5-P_x/6$<br>S2-3	$P_f/5-P_x/6$<br>S1-1	$P_f/5-P_x/6$<br>S1-2	$P_f/5-P_x/6$<br>S1-3	$P_x/3$<br>S2-1	$P_x/3$<br>S2-2	$P_x/3$<br>S2-3	0	0	0
	S2-2	$P_f/5-P_x/6$<br>S2-1	$1-P_f-P_x/6$<br>S2-2	$P_f/5-P_x/6$<br>S2-3	$P_f/5-P_x/6$<br>S1-1	$P_f/5-P_x/6$<br>S1-2	$P_f/5-P_x/6$<br>S1-3	$P_x/3$<br>S2-1	$P_x/3$<br>S2-2	$P_x/3$<br>S2-3	0	0	0
	S2-3	$P_f/5-P_x/6$<br>S2-1	$P_f/5-P_x/6$<br>S2-2	$1-P_f-P_x/6$<br>S2-3	$P_f/5-P_x/6$<br>S1-1	$P_f/5-P_x/6$<br>S1-2	$P_f/5-P_x/6$<br>S1-3	$P_x/3$<br>S2-1	$P_x/3$<br>S2-2	$P_x/3$<br>S2-3	0	0	0
	S1-1	$P_f/5-P_x/6$<br>S2-1	$P_f/5-P_x/6$<br>S2-2	$P_f/5-P_x/6$<br>S2-3	$1-P_f-P_x/6$<br>S1-1	$P_f/5-P_x/6$<br>S1-2	$P_f/5-P_x/6$<br>S1-3	$P_x/3$<br>S2-1	$P_x/3$<br>S2-2	$P_x/3$<br>S2-3	0	0	0
	S1-2	$P_f/5-P_x/6$<br>S2-1	$P_f/5-P_x/6$<br>S2-2	$P_f/5-P_x/6$<br>S2-3	$P_f/5-P_x/6$<br>S1-1	$1-P_f-P_x/6$<br>S1-2	$P_f/5$<br>S1-3-<br>$P_x/6$	$P_x/3$<br>S2-1	$P_x/3$<br>S2-2	$P_x/3$<br>S2-3	0	0	0
	S1-3	$P_f/5-P_x/6$<br>S2-1	$P_f/5-P_x/6$<br>S2-2	$P_f/5-P_x/6$<br>S2-3	$P_f/5-P_x/6$<br>S1-1	$P_f/5-P_x/6$<br>S1-2	$1-P_f-P_x/6$<br>S1-3	$P_x/3$<br>S2-1	$P_x/3$<br>S2-2	$P_x/3$<br>S2-3	0	0	0

The XRPD patterns were simulated by using the DIFFAX routine operated in recursive mode.[23] A wavelength of 0.180872 Å was applied, which corresponds to the wavelengths of the synchrotron XRPD measurements. A Gaussian function was used to approximate diffraction line broadening caused by the instrumental function as well as by a finite (ca. 200 nm) crystallite size. All patterns were simulated in a 2θ range between 0.1 ° and 34.0 ° with a step size of 0.005 °. All faulting scenarios were used for the simulations with transition probabilities given in Table SI-6.

Table SI-6 Applied microstructural parameters for the simulations of the XRPD patterns of stacking faulted TTI-COF.

Applied Faulting Scenario	Transition Probability Matrix	Used microstructural parameters
Faulting Scenario 1	Table SI-1	$P_f = 2/3$
Faulting Scenario 2	Table SI-2	$P_f = 1/2$
Faulting Scenario 3	Table SI-3	$P_f = 5/6$
Faulting Scenario 4	Table SI-4	$P_f = 1/2$
Faulting Scenario 5	Table SI-5	$P_f = 5/6$, $P_f = 1/3$

All refinements of the XRPD patterns were carried out by using the TOPAS software,[5] and the DIFFAX-like supercell approach.[24] For each refinement the calculated diffraction patterns of 300 supercells containing 200 layers each were averaged. As an optimization of the microstructural parameters, i.e., of the transition probabilities, is not possible by a classical least square approach, a grid search optimization was performed.[25,26] For the refinement of the measured diffraction pattern of LT, faulting scenario 3 (Table SI-3, Figure SI-14) was used, which fits the observed pattern best, while conforming to antiparallel stacking. The fault probability P_f was varied from 0.00 to 1.00 in increments of 0.01.

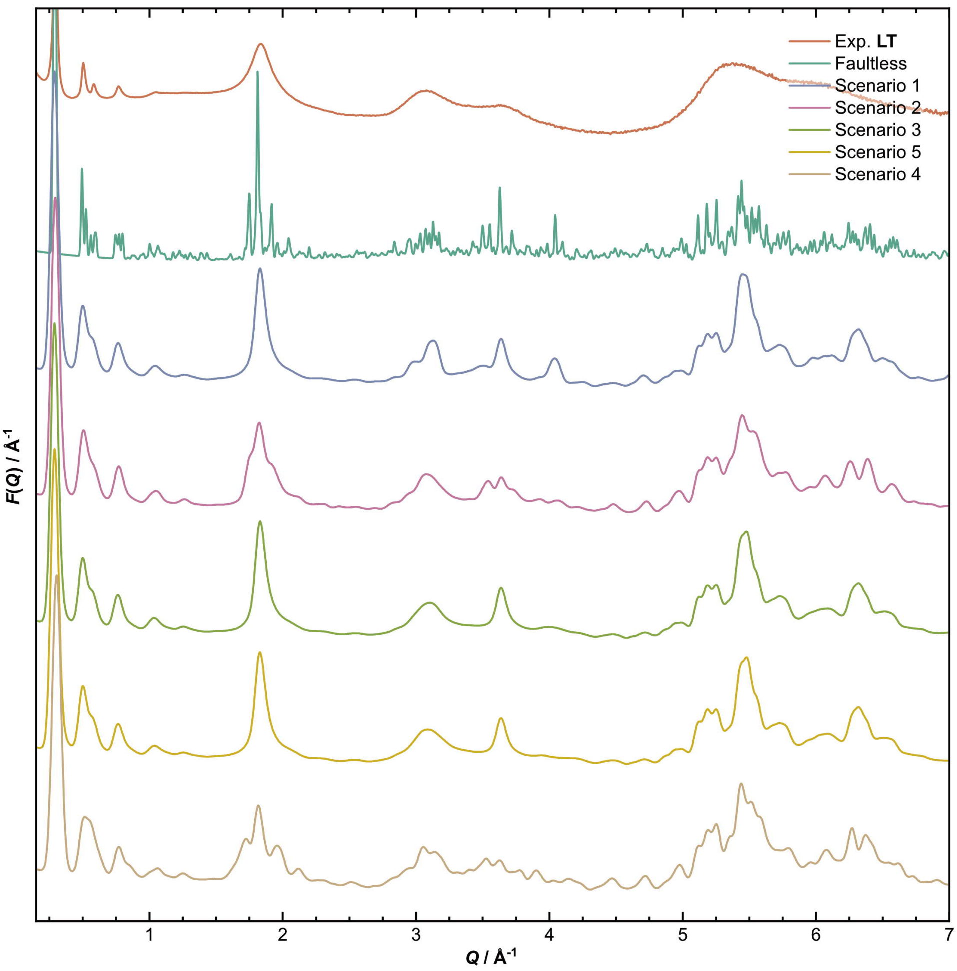

Figure SI-17 Simulated total scattering structure functions for the five stacking fault scenarios as compared to the experimental data collected for LT. Scenarios 2, 3, 4, and 5, but not scenario 1 or the faultless pattern, gave simulated F(Q) that could reasonably describe the measured data, especially in the range of 2.8 $Å^{-1} < Q < 4$ $Å^{-1}$.

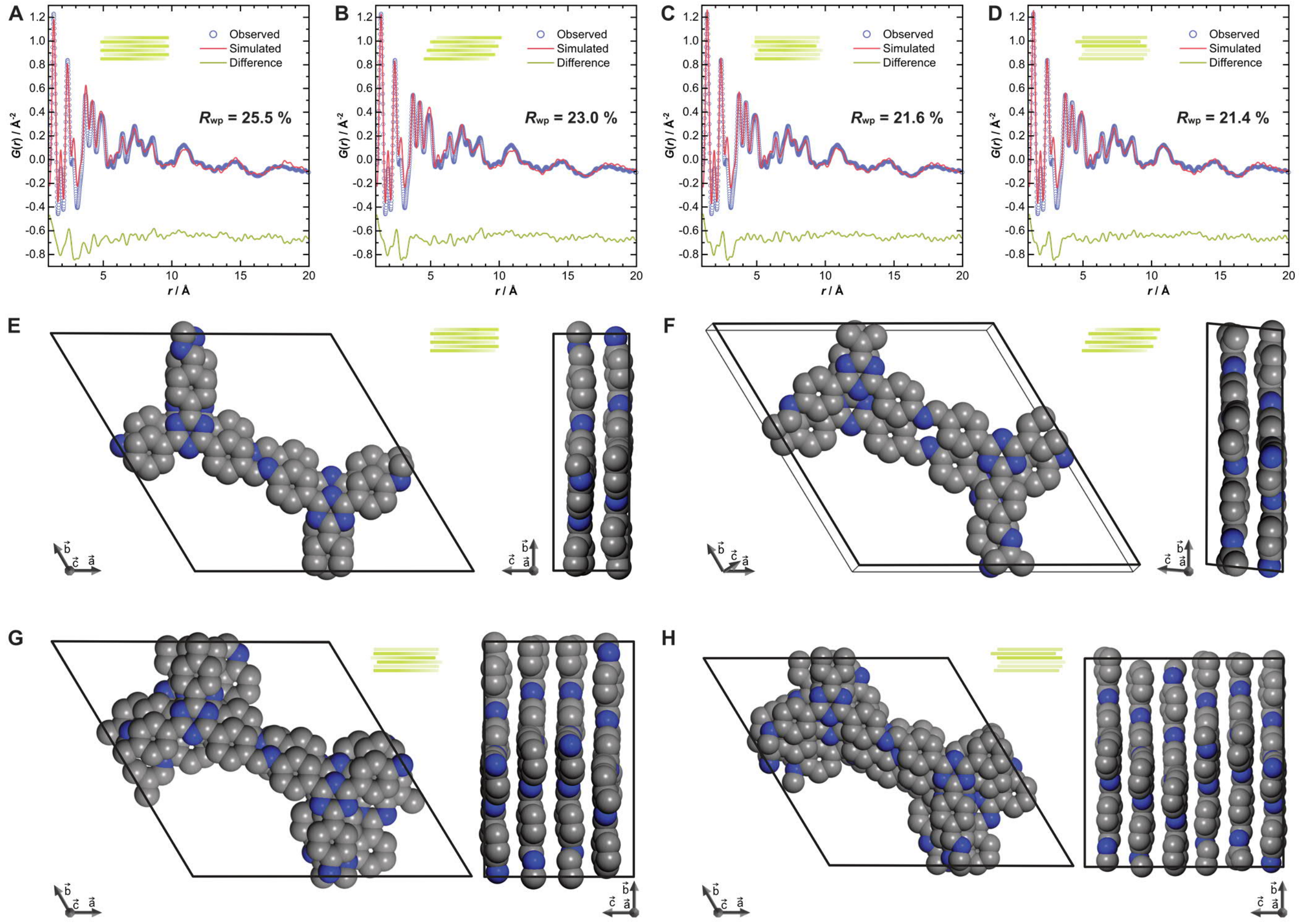

Figure SI-18 (A) Best obtained PDF fits over 1 Å–20 Å from structure refinements of LT, assuming antiparallel stacking with one layer translated, (B) antiparallel stacking with one layer translated and α and β refined, (C) antiparallel stacking with unit cell doubled in [001] direction and three layers translated, and (D) antiparallel stacking with unit cell tripled in [001] direction and five layers translated. (E) View onto and across unit cells obtained from PDF fits of LT assuming antiparallel stacking with one layer translated, (F) antiparallel stacking with one layer translated and α and β refined, (G) antiparallel stacking with unit cell doubled in [001] direction and three layers translated, and (H) antiparallel stacking with unit cell tripled in [001] direction and five layers translated.

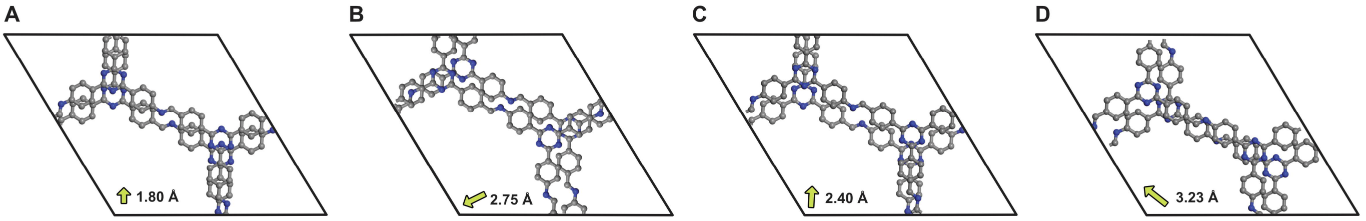

Figure SI-19 (A) Unit cell derived by PDF refinement, assuming antiparallel eclipsed stacking with one layer translated. In-plane offset vector indicated. (B-D) Two-layer sections from unit cell derived by PDF refinement, assuming antiparallel eclipsed stacking with unit cell tripled in [001] direction and five layers translated. In-plane offset vectors indicated.

Additional data from Diamond Light Source

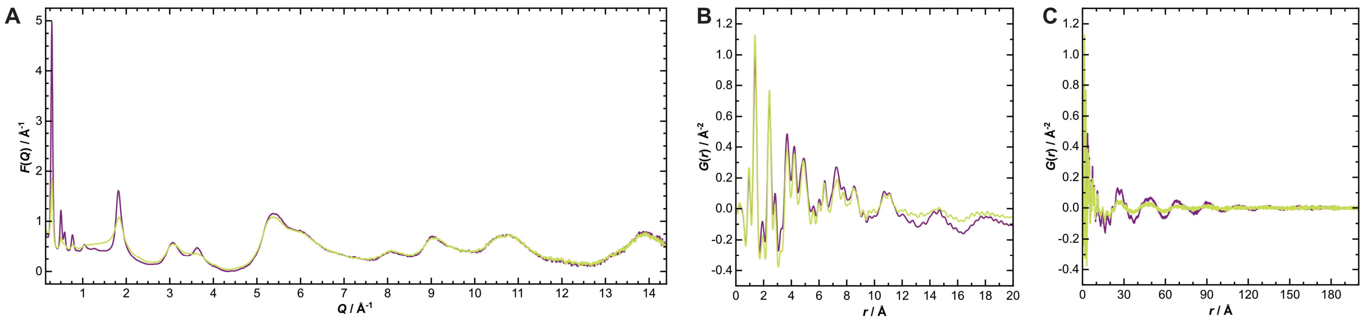

Figure SI-20 (A) Comparison of reduced total scattering structure functions F(Q) for HT (purple) and LT (green) collected at Diamond Light Source. (B) Pair distribution functions derived thereof up to 20 Å and (C) up to 200 Å.

Summary of lattice parameters

Table SI-7 Overview of lattice parameters obtained by Rietveld or PDF fits. Standard deviation given in parentheses. All refinements were performed in P1 space group. Parameters in italics were not refined.

Technique	Sample	Structure motif	R_{wp} (in %)	Lattice parameters (in Å or °) a	b	c	α	β	γ	Interlayer distance (in Å)	No. of layers	Interlayer offset (in Å)
Rietveld	HT	Antiparallel, eclipsed	9.46	25.08531(2377)		6.98876(6930)	90.0	90.0	120.0	3.494	2	0,0
	HT	Antiparallel, unidirectionally slipped	4.18	25.78103(1978)		7.27192(1934)	80.83014(875)		120.0	3.446	2	1.867(25), 3.104(21)[a]
	LT	Antiparallel, eclipsed	3.74	24.95532(2127)		6.93367(9159)	90.0	90.0	120.0	3.467	2	0,0
	LT	Antiparallel, unidirectionally slipped	3.36	25.34074(19597)		7.06838(8953)	83.17049(16838)		120.0	3.433	2	2.649(28), 2.239(19)[a]
	LT	Supercell, best refinement	2.70	–	–	–	–	–	–	–	200	–
PDF	HT	Antiparallel, eclipsed	21.8	26.1882(1.4)	25.3498(1.1)	7.02967(0.29)	90.0	90.0	121.257	3.515	2	0,0
	HT	Antiparallel, unidirectionally slipped	12.1	25.9102(1.7)	25.83930(1.7)	7.21198(0.34)	81.1168(16)	83.0649(12)	121.515(4.2)	3.459	2	2.425(23), 1.219(43)[a]
	HT	Parallel, eclipsed	23.1	26.0843(1.6)	25.3455(1.3)	3.51765(0.14)	90.0	90.0	121.257	3.518	1	0,0
	HT	Parallel, unidirectionally slipped	13.6	25.6384(1.3)	25.9674(1.7)	3.6096(0.16)	76.8759(10)	90.2122(12)	121.187(4.4)	3.483	1	0.949(0)[a]
	LT	Antiparallel, eclipsed	32.0	25.8083(1.3)	25.5915(1.3)	7.14492(0.19)	90.0	90.0	121.257	3.572	2	0,0
	LT	Antiparallel, unidirectionally slipped	26.6	25.7849(1.3)	25.8419(1.2)	7.33192(0.22)	79.5791(6.1)	82.4008(7)	121.684(2.7)	3.470	2	2.568(23), 1.200(43)[a]
	LT	Parallel, eclipsed	38.6	26.0489(0.62)	25.1823(0.7)	3.5617(0.076)	90.0	90.0	121.257	3.562	1	0,0
	LT	Parallel, unidirectionally slipped	28.9	25.6566(0.71)	25.652(1.2)	3.64915(0.1)	77.9715(9.6)	88.6814(13)	120.687(3.2)	3.527	1	0.936(0)[a]
	LT	Antiparallel, 1 layer translated	25.5	25.8301(0.94)	25.6528(0.95)	7.03011(0.17)	90.0	90.0	121.257	3.515	2	1.80(0)[b]
	LT	Antiparallel, 1 layer translated	23.0	25.6499(1.1)	25.7973(0.98)	7.03464(0.17)	88.2632(9.8)	84.5022(12)	121.257	3.486	2	3.217(21), 2.648(16)[a]
	LT	Antiparallel, 3 layers translated	21.6	25.9052(0.91)	25.5427(0.95)	13.8731(0.34)	90.0	90.0	121.257	3.468	4	3.25(158)[b]
	LT	Antiparallel, 5 layers translated	21.4	25.8144(1.1)	25.6449(1.1)	20.7899(0.5)	90.0	90.0	121.257	3.465	6	3.28(132)[b]
PDF simplified unit cell	HT	Layer-to-layer	27.1	–[c]		6.92278 (0.15)	90.0	90.0	120.0	3.461	2	0
	LT	Layer-to-layer	48.7	–[c]		6.84891 (0.59)	90.0	90.0	120.0	3.424	2	0
	HT	Pore-to-pore	18.1	25.5313(15)[d]		–[e]	85.166(79)	82.4865(66)	120.738(30)	–[e]	–	–
	LT	Pore-to-pore	28.4	25.2357(19)[d]		–[e]	85.1134 (150)	82.3947(110)	120.266(60)	–[e]	–	–

Uncertainties are quoted as given by the respective refinement programs. a: Calculated from four measured interatom distances for each pair of neighboring layers. b: Average, calculated from refined layer offsets in directions [100] and [010] for each pair of neighboring layers. c: Approximate unit cell parameters for graphite were used but not refined. d: Average value of a and b. e: Cannot be refined here, since the stacking component barely contributes to the PDF signal for r > 20 Å.

References

1 S. Hayashi and K. Hayamizu, Bull. Chem. Soc. Jpn., 1991, 64, 685–687.

2 S. Hayashi and K. Hayamizu, Bull. Chem. Soc. Jpn., 1991, 64, 688–690.

3 M. Thommes, K. Kaneko, A. V. Neimark, J. P. Olivier, F. Rodriguez-Reinoso, J. Rouquerol and K. S. W. Sing, Pure Appl. Chem., 2015, 87, 1051–1069.

4 DFT models library of DFT and GCMC methods in Quantachrome's data reduction software, https://www.quantachrome.com/technical/dft.html, (accessed 30 April 2020).

5 A. A. Coelho, J. Appl. Crystallogr., 2018, 51, 210–218.

6 P. J. Chupas, X. Qiu, J. C. Hanson, P. L. Lee, C. P. Grey and S. J. L. Billinge, J. Appl. Crystallogr., 2003, 36, 1342–1347.

7 G. Ashiotis, A. Deschildre, Z. Nawaz, J. P. Wright, D. Karkoulis, F. E. Picca and J. Kieffer, J. Appl. Crystallogr., 2015, 48, 510–519.

8 C. J. Wright and X. D. Zhou, J. Synchrotron Radiat., 2017, 24, 506–508.

9 GitHub - xpdAcq/xpdtools: Stand alone data processing tools, https://github.com/xpdAcq/xpdtools, (accessed 20 May 2020).

10 M. Basham, J. Filik, M. T. Wharmby, P. C. Y. Chang, B. El Kassaby, M. Gerring, J. Aishima, K. Levik, B. C. A. Pulford, I. Sikharulidze, D. Sneddon, M. Webber, S. S. Dhesi, F. Maccherozzi, O. Svensson, S. Brockhauser, G. Náray and A. W. Ashton, J. Synchrotron Radiat., 2015, 22, 853–858.

11 E. Takeshi and S. J. L. Billinge, Underneath the Bragg Peaks: Structural Analysis of Complex Materials, Pergamon, 2nd edn., 2012, vol. 16.

12 S. J. L. Billinge, in Int. Tabl. Cryst. Vol. H, eds. C. J. Gilmore, J. A. Kaduk and H. Schenk, International Union of Crystallography, Chester, England, 2019.

13 P. Juhás, T. Davis, C. L. Farrow and S. J. L. Billinge, J. Appl. Crystallogr., 2013, 46, 560–566.

14 P. F. Peterson, E. S. Božin, T. Proffen and S. J. L. Billinge, J. Appl. Crystallogr., 2003, 36, 53–64.

15 S. J. L. Billinge and C. L. Farrow, J. Phys. Condens. Matter, , DOI:10.1088/0953-8984/25/45/454202.

16 X. Yang, P. Juhas, C. L. Farrow and S. J. L. Billinge, arXiv Prepr., 2014, arXiv:1402.3163.

17 C. L. Farrow, P. Juhas, J. W. Liu, D. Bryndin, E. S. Božin, J. Bloch, T. Proffen and S. J. L. Billinge, J. Phys. Condens. Matter, 2007, 19, 335219.

18 S. Brunauer, P. H. Emmett and E. Teller, J. Am. Chem. Soc., 1938, 60, 309–319.

19 M. Thommes, Chemie-Ingenieur-Technik, 2010, 82, 1059–1073.

20 F. Haase, K. Gottschling, L. Stegbauer, L. S. Germann, R. Gutzler, V. Duppel, V. S. Vyas, K. Kern, R. E. Dinnebier and B. V. Lotsch, Mater. Chem. Front., 2017, 1, 1354–1361.

21 Q. Mou, C. J. Benmore and J. L. Yarger, J. Appl. Crystallogr., 2015, 48, 950–952.

22 H. Schlomberg, J. Kröger, G. Savasci, M. W. Terban, S. Bette, I. Moudrakovski, V. Duppel, F. Podjaski, R. Siegel, J. Senker, R. E. Dinnebier, C. Ochsenfeld and B. V. Lotsch, Chem. Mater., 2019, 31, 7478–7486.

23 M. M. J. Treacy, J. M. Newsam and M. W. Deem, Proc. - R. Soc. London, A, 1991, 433, 499–520.

24 A. A. Coelho, J. S. O. Evans and J. W. Lewis, J. Appl. Crystallogr., 2016, 49, 1740–1749.

25 S. Bette, T. Takayama, V. Duppel, A. Poulain, H. Takagi and R. E. Dinnebier, Dalt. Trans., 2019, 48, 9250–9259.

26 S. Bette, B. Hinrichsen, D. Pfister and E. Dinnebier, Robert, J. Appl. Crystallogr., 2020, 53, 76–87.
